# The failure of DAC to induce OCT2 expression and its remission by hemoglobin-based nanocarriers under hypoxia in renal cell carcinoma

**DOI:** 10.7150/thno.39944

**Published:** 2020-02-18

**Authors:** Lu Chen, Zeyang Wang, Qingwen Xu, Yuxi Liu, Le Chen, Suhang Guo, Hua Wang, Kui Zeng, Junqing Liu, Su Zeng, Lushan Yu

**Affiliations:** 1 Institute of Drug Metabolism and Pharmaceutical Analysis, Zhejiang Province Key Laboratory of Anti-Cancer Drug Research, College of Pharmaceutical Sciences, Zhejiang University, Hangzhou 310058, China.; 2 Department of Urology, Cancer Hospital of Zhejiang Province, Hangzhou 310022, China; 3 The First Affiliated Hospital, School of Medicine, Zhejiang University, Hangzhou 310022, China

**Keywords:** renal cell carcinoma, hypoxia, OCT2, ENT1, nanoparticles

## Abstract

**Background:** Human organic cation transporter 2 (OCT2) is the most abundant and important uptake transporter involved in the renal excretion of cationic drugs. Abnormal hypermethylation- mediated silencing of OCT2 results in oxaliplatin resistance in renal cell carcinoma (RCC). The epigenetic activation of OCT2 by decitabine (DAC) reversed this resistance in normoxic conditions. Given the hypoxic characteristic of RCC, it is still unclear whether hypoxia promotes DAC resistance and is involved in the regulation of OCT2.

**Methods:** The mRNA and protein expression of OCT2 was determined by qRT-PCR and Western blotting. MSRE-qPCR and BSP were used to examine methylation modifications at the OCT2 promoter. The ChIP-qPCR analysis was performed to detect the abundance of histone modification and HIF-1α. The accumulation of DAC and 5-mC were detected using LC-MS, and the amount of 5-hmC was determined by dot blot analysis. To understand the role of hypoxia in the regulation of equilibrative nucleoside transporter 1 (ENT1) expression, the HIF-1α KO cell model was constructed. The re-emulsion method was used for the construction of H-NPs, an oxygen nanocarrier based on hemoglobin, to alleviate the drug resistance of DAC under hypoxia.

**Results:** DAC was unable to upregulate OCT2 expression in hypoxic conditions because of the hypermethylation and low H3K4me3 modification in its promoter region. Hypoxia-mediated repression of human ENT1, which was markedly suppressed in RCC, resulted in a decrease in the cellular accumulation of DAC. Besides, hypoxia-induced upregulation of histone deacetylase HDAC9, which impaired the enrichment of H3K27ac modification in the OCT2 promoter, led to the transcriptional repression of OCT2. H-NPs could attenuate the hypoxia-induced loss of DAC activity and sensitize RCC cells to the sequential combination therapy of DAC and oxaliplatin.

**Conclusions:** Hypoxia-mediated repression of ENT1 led to the inability of DAC to upregulate the expression of OCT2 under hypoxia. H-NPs could alleviate resistance to oxaliplatin and DAC in RCC cells under hypoxia and may have potential clinical applications.

## Introduction

Renal cell carcinoma (RCC) is the most lethal type of urinary tract cancer, with an incidence of approximately 3.8% and accounts for 2.5% of all mortalities related to adult malignancies 1. Among all subtypes, clear cell renal cell carcinoma (ccRCC) accounts for 70%-75% of RCCs 2, which are insensitive to radiotherapy. Only 10%-15% of chemotherapy regimens for metastatic renal cell carcinoma (mRCC) are effective 3. Patients undergoing targeted therapies or immunotherapies, the current clinical practices in the treatment of mRCC, may still experience serious adverse reactions or acquired resistance 4-7. Therefore, there is an urgent need to explore and develop new targets or combination drug therapies with reduced toxicity and better efficacy. Human organic cation transporter 2 (OCT2, encoded by *SLC22A2*) is the most abundant and important uptake transporter in the renal excretion process of cationic drugs. In a previous study, we reported that abnormal hypermethylation-mediated silencing of OCT2 resulted in the resistance of renal cancer cells to oxaliplatin and platinum-based chemotherapeutic agents 8, 9.

DNA methylation refers to the addition of a methyl group at the C-5 position of cytosine (5-mC), predominantly within CpG dinucleotides, by maintenance or *de novo* DNA methyltransferases (mainly DNMT1, DNMT3A, and DNMT3B) 10. Hypermethylation in distal or proximal gene regulatory elements, including transcriptional start sites (TSS) and enhancers, is often associated with the transcriptional repression of nearby genes 11. In the mammalian lifecycle, DNA methylation can be dynamically regulated by active DNA demethylation pathways, including the ten-eleven-translocation (TET) enzyme-mediated stepwise oxidation of 5-mC, activation-induced deaminase (AID), DNA glycosylase (TDG)-mediated deamination of 5-mC to thymine coupled with base excision repair, or passive DNA demethylation pathways (inhibition of DNMTs)^12^. Previous studies have shown that the combination of decitabine (DAC), an inhibitor of DNMTs, with the traditional chemotherapy drug oxaliplatin yielded satisfactory anticancer effects both *in vitro* and *in vivo* experiments by reversal of hypermethylation in the promoter of *SLC22A2,* thereby increasing the expression of OCT2 in renal cancer cells 8, 9.

A hypoxic microenvironment is found in up to 50%-60% of solid tumors 13 and is typically associated with abnormal vasculature and increased oxygen consumption by cancer cells 14. Hypoxia induces the stabilization of hypoxia-inducible factors (HIF), which exerts a pivotal role in tumor progression through regulating the expression of downstream target genes involved in proliferation, apoptosis, angiogenesis, genetic instability, tumor metabolism, and immune response 15. Hypoxic tumors exhibit enhanced resistance to radiation and chemotherapy because of the inhibition of apoptosis or senescence and the activation of drug efflux or cellular metabolism 16. Hypoxic tumor cells also display a distinct epigenetic profile 17. Evidence has accumulated showing that hypoxia can lead to the downregulation of DNMTs 18, render the oxygen-dependent TET enzymes 19 or histone demethylation inactive 20, 21, influence the levels of histone methylation and acetylation 17, 22, and regulate the expression of non-coding RNAs 23. Oxygen concentration in normal kidney tissues is 9.5% 21, but RCC, as a solid tumor, exhibits a hypoxic tumor microenvironment. Although the sequential combination therapy of DAC and oxaliplatin exerts a remarkable antitumor effect, it is still unclear if this therapeutic scheme is effective in a hypoxic environment and if hypoxia is involved in the regulation of OCT2.

In this study, we have investigated the demethylating effect of DAC in a hypoxic environment and investigated the mechanisms underlying hypoxia-mediated DAC resistance and OCT2 repression in RCC. Furthermore, we developed a simple and efficient oxygen nanocarrier based on hemoglobin, H-NPs, in combination with DAC to alleviate hypoxia-mediated drug resistance of oxaliplatin and DAC in RCC.

## Methods

### Cell culture and Drug Treatment

RCC cell lines (786-O, 769-P, Caki-1-1, ACHN, RCC4) and HEK-293T cell lines were obtained from the Chinese Academy of Science Committee on type culture collection cell libraries. Cell lines were maintained following instructions from ATCC. The hypoxia cell model was cultured in anaerobic incubator (Electrotek AW400SG, England) at 1% oxygen concentration. The three-dimensional cell sphere was cultured in DMEM/F-12 containing 2 mM/L glutamine, 100 U/mL penicillin and streptomycin, 1×B27, 20 ng/mL EGF, 10 ng/mL FGF in ultra-low adherent petri dish (Corning).

For decitabine (Sigma-Aldrich), NBTI (Sigma-Aldrich) and DMOG (D136754, Aladdin) treatment, cells were seeded into 6-well plates and pre-cultured to 10%~20% confluency. Indicated doses of drug were added to cell culture medium, which was refreshed daily.

### Patient samples collection

Human matched renal normal-tumor surgical samples were collected from 40 renal cell carcinoma patients in Specimen Bank of Zhejiang Cancer Hospital (Hangzhou, China), with the approval by the Institutional Review Board of Zhejiang Cancer Hospital (No. 2014-08-76). Relevant pathological information were summarize in supplementary Table S3.

### Animals

Six-week-old male BALB/c-nu mice were ordered from Shanghai slack laboratory animal co., LTD China. All animal protocols followed institutional guidelines and were approved by Zhejiang University Animal Care and Use Committee.

### RNA extraction and quantitative real-time PCR

Total RNA from cell lines was isolated with a Axygen total RNA mini-prep kit, while total RNA from tissue samples was isolated by a total RNA mini-prep kit (Tiangen). Then PrimeScript® RT Master Mix (DRR036A, Takara) was used to reverse transcribe 500 ng RNA. Quantitative real time PCR was performed with SYBR ® Premix EX Taq (Takara) in StepOnePlus Real-Time PCR System (StepOnePlus) and ΔΔCt method was used for gene expression analysis with *GAPDH* as internal control. Primers used in this study were listed in supplementary Table S1.

### Western blot analysis

Cells were harvested and lysed using RIPA buffer (Beyotime) supplemented with PMSF (Beyotime), leupeptin (Beyotime) and pepstatin (Beyotime). The membrane proteins were extracted by Mem-PER™ Plus Membrane Protein Extraction kit (Thermo Fisher, USA). The protein concentrations of lysate were determined by bicinchoninic acid (BCA) assay. Protein extracvts were subjected to SDS-PAGE analysis and transferred to polyvinylidene difluoride (PVDF) membrane after electrophoresis. The membranes were blocked with 5% BSA buffer followed by antibody hybridization and then visualized in G-BOX gel imaging system (Chemi XR 5, Syngene). Antibodies and dilution ratio in this study include anti-OCT2 (HPA008567, Sigma-Aldrich) at 1:200 dilution, anti-GAPDH (KC-5G4, Kangchen) at 1:5000 dilution, anti-ATP2A2 (AB54032, Sangon Biotech) at 1:5000 dilution, anti-ENT1 (11337-1-AP, proteintech) at 1:1000 dilution, anti-HIF-1α (610958, BD Biosciences), (NB100-105, Novus) at 1:800 dilution, anti-HIF-2α (NB100-122, Novus) at 1:1000 dilution, anti-H3K27ac (ab4729, Abcam) at 1:3000 dilution, anti-H3K18Ac (ab1191, Abcam) at 1:3000 dilution and anti-H3 (ab1791, Abcam) at 1:3000 dilution.

### Dot blot analysis

Genomic DNA was isolated with QIAamp® DNA Mini Kit (Qiagen) and quantified by NanoDrop 2000 then diluted to the same concentration with ddH2O. The extracted DNA was then added with 4 M NaOH and incubated at 95 °C for 10 min, cooled on ice. 1 μL of the sample was dropped on nitrocellulose membranes carefully. Each side did UV irradiation (2000 w) for 15 min. The crosslinked nylon membrane was placed in a blocking solution (5% BSA) at room temperature for 1 h, followed by anti-5-hmC (1:1000) hybridization, and then incubated with secondary antibody (1:5000) for 2 h. Finally, G-BOX gel imaging system (Chemi XR 5, Syngene) was used for densitometry analysis.

### Immunohistochemistry (IHC)

Human matched renal normal-tumour surgical samples were collected and fixed in formaldehyde immediately. Subsequent histological experiments were undertaken by Wuhan Servicebio Technology Co., Ltd. Anti-ENT1 (11337-1-AP, proteintech) was used for immunofluorescence staining at 1:200 dilution. The results of IHC were viewed and analyzed in Case Viewer software.

### Bisulfite Sequencing Analysis

BSP experiments were carried out as previously described^8^. Bisulfite specific primers used in this study were listed in supplementary Table S1. At least 10 clones for each sample were sequenced analyzed.

### Methylation-sensitive restriction enzymes-qPCR (MSRE-qPCR)

MSRE-qPCR experiments were carried out as previously described^8^ with minor modification. Briefly, Genomic DNA was isolated with QIAamp® DNA Mini Kit (Qiagen). 200 ng DNA of each group was used for restriction endonuclease reaction (Pmil+Xbal+/Pmil-Xbal+) 37 °C water bath for 2 h and terminated at 80 °C for 10 min. The products were analyzed by quantitative real-time PCR (primers were listed in supplementary Table S1). The E-Box methylation was calculated by the following equation:

Methylated E Box%=2^-(CtPmiI+XbaI-Ct XbaI)^ ×100%。

### Analysis of global 5-mC levels in cultured cells

RCC cells were seeded into 24-well plates. 48 h later, cells were washed with 200 μL Hanks Balanced Salt Solutions (HBSS) twice, followed by 100 μL sodium dodecyl sulfonate (SDS, Sigma) to lyse cells. After repeated pipetting for more than 20 times, all the lysates were transferred to a 1.5 mL centrifuge tube, then centrifuge at 13,000 rpm for 15 min at room temperature. Transfer 40 μL of the supernatant to a new centrifuge tube, add 80 μL of acetonitrile precipitated protein containing 50 ng/mL internal standard (deuterated isotope of cytosine) , vortex for 15 s, centrifuge at room temperature for 15 min at 13,000 rpm. Remove 100 μL of supernatant for further determination.

Quantitative detection of cytosine and methylcytosine was performed on Waters Xevo TQD (Waters, USA), with detailed parameters below:

Chromatographic conditions: TSKGel Amide-80 (2.0 mm × 150 mm, particle size 5 μm) was used for 5-mC detection. Phase A is pure water and Phase B is methanol containing 0.1% formic acid. The flow rate was 0.300 mL/min, the injection volume was 7.0 μL, and the autosampler temperature was 10 °C. Isocratic elution with elution conditions of 30% A, 70% B.

Mass spectrometry conditions: ESI^+^, ion source temperature 140 °C, desolvation temperature: 350 °C, capillary voltage: 2.5 kV, cone voltage: 30 V, collision energy: 20 V, collision gas velocity: 0.15 mL/min, desolvation gas flow rate was 1000 L/h, the cone gas flow rate was 150 L/h, MRM monitoring mode.

Quantitative ion pair: cytosine *m/z* 112→95; methylcytosine *m/z* 126→109; cytosine isotope *m/z* 115→98.

### Cellular uptake assays

Entecavir (ETV) provided by Meilun Biologic Co., Ltd. (Dalian, China) is a substrate of ENT1 and was applied to determine the transport activity of ENT1. The quantification of ETV in cell lysates was detected by an Agilent 1290-6460 liquid chromatography-mass spectrometer with a triple quadrupole mass spectrometer (Agilent, Santa Clara, CA) with the method established in our laboratory before.

The accumulation of DAC was detected using LC-MS by Agilent 1290-6460. RCC cells were incubated in HBSS containing 500 μM DAC or ENT1 inhibitor, NBTI (Sigma), at 37 °C for 3 min after rinsed 3 times with pre-warmed HBSS. 1 ng/mL loratadine (Aladdin) was used as internal standard. Chromatographic conditions of DAC detection was same to 5-mC as described in 2.10.

Mass spectrometry conditions: ESI^+^, ion source temperature: 140 °C, desolvation temperature: 350 °C, capillary voltage: 3.5 kV; cone voltage: 500 V, gasification gas flow: 5 L/min, spray pressure: 45 psi, sheath gas temperature was 350 °C, the sheath gas flow rate was 11 L/min.

Quantitative ion pair: DAC *m/z* 251→135, fragmentation voltage and collision energy were 100 V and 4 V, respectively; loratadine (IS) *m/z* 383→337, fragmentation voltage and collision energy were 170 V and 20 V, respectively.

### Construction of ENT1 stablely expressed cell model

ENT1 stable expressed 769-P cell lines were constructed for further research. PCDH-ENT1 plasmid was constructed for lentiviral transfection. Cells were performed qRT-PCR, western-blot and uptake assays to ensure the transport function of ENT1 stable expressed 769-P (termed pCDH-ENT1 ).

### Chromatin Immunoprecipitation (ChIP) Assay

ChIP experiments were performed as previously described^8^. Primers used in ChIP-qPCR are listed in supplementary Table S1. ChIP grade antibodies, used in this study, including anti-HIF-1α (610958, BD Biosciences), anti-HIF-1α (NB100-105, Novus), anti-H3 (ab1791, Abcam), Anti-RNA polymerase II (ab5408, Abcam), anti-H3K27ac (ab4729, Abcam) and normal rabbit IgG (sc-2027, Santa Cruz Biotechnology). Enrichment was normalized to total input.

### Preparation of H-NPs, F-D-H-NPs, and ICG-H-NPs

The re-emulsion method was used for the construction of H-NPs. Briefly, 400 μL of hemoglobin (Bovine, Sinopharm) solution was placed in a 10 mL centrifuge tube, added 2 mL oil phase (PLGA solution, Sigma) was added and mixed using a whirlpool oscillator ice bath ultrasound for 3 minutes in the ultrasonic breaker to form colostrum. After complete emulsification, 1% of PVA and F68 solution (70:30, v/v) was added and subjected to low-temp ultrasound for 5 min to re-form the emulsion. A decompression rotary evaporator was used to remove the organic solvents, and the mixture was centrifuged at 10,000 rpm for 10 min. Subsequently, the supernatant was removed, the pellet was washed with distilled water 3 times, freeze-dried, and re-dissolved in deionized water to form PLGA-HB-NPs. The chitosan was dissolved () stirred by for 20 min. The obtained PLGA-HB-NPs solution was added dropwise into 1 mg/mL chitosan solution in deionized water and incubated for 12 h at 4 °C with magnetic stirring. The solution was centrifuged for 30 min at 12,000 rpm, then for 6 min at 15,000 rpm and finally filtered through 0.45 μm membrane. Oxygen carriers were charged with oxygen in the medium for 3 s before use.

The Fluorescein isocyanate (FITC)-stained chitosan was prepared as described in the previous report 24. In brief, 200 mg chitosan solution was dissolved in 20 mL 0.1 M acetic acid to which 10 mL FITC solution (including 2 mg/mL methanol) was added, stirred in the darkroom for 3 h, and pH was adjusted to 8.0-9.0 by 0.5 M NaOH solution. The mixture was centrifuged for 15 minutes at 20,000 rpm and the supernatant was removed, washed with deionized water, re-dissolved in 20 mL 0.1 M acetic acid solution, and dialyzed (10-12 kDA) in deionized water for 3 days in the darkroom. The FITC-chitosan was then examined by fluorescence microscopy (excitation wavelength 495 nm). Next, 1,1'-dioctadecyl-3,3,3',3'-tetramethylindocarbocyanine perchlorate (Dil) solution 2 μg/mL in DMSO was crosslinked to chitosan. The preparation process of the FITC and Dil double-stained H-NPs (F-D-H-NPs) was the same as H-NPs. The chitosan was pre-stained by indocyanine green (ICG). Briefly, ICG, EDC, and DMAP (ICG/EDC/DMAP - 1:3:3 mol/mol/mol) were reacted for 1 h at 45 °C in aqueous solution. Chitosan was then added to the solution with stirring for 24 h. The solution was centrifugated and rinsed with deionized water repeatedly to remove the unreacted ICG. The ICG-chitosan was used to prepare the ICG-H-NPs the preparation process of which was the same as F-D-H-NPs.

### Characterization of H-NPs

The particle size distribution and Zeta-potential of H-NPs were measured by the dynamic light scattering instrument (Malvern, UK). H-NPs were observed by Transmission Electron Microscopy (TEM) instrument (HITACHI) and pre-negative staining by 2% phosphotungstic acid (PTA) was performed. The uv-vis spectra of oxy/deoxyhemoglobin- and oxygen- loaded/unloaded H-NPs were measured by ultraviolet-visible spectrophotometer (Persee).

### The loading efficiency (LE) and encapsulation efficiency (EE) of nanocarriers

The Loading efficiency (LE) and encapsulation efficiency (EE) of the nanocarriers were measured by bicinchoninic acid (BCA) assay (Beyotime). Tested by automatic microplate reader (molecular devices) at BCA module. The calculations were based on the following equations.

Loading efficiency(LE)=

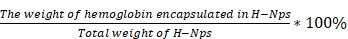
 (1)

Encapsulation efficiency (EE)=

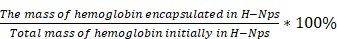
 (2)

### Platinum quantification by ICP-MS

Cells were incubated at 37 °C in the presence of 100 μM oxaliplatin (Sigma) for 48 h. Cells were washed twice with ice cold PBS and collected by centrifugation. A small amount of cell pellet was taken for cell counting. The amount of platinum in samples was determined by inductively coupled plasma mass spectrometry (ICP-MS, Hangzhou Leading Pharmatech Co.,Ltd, China).

### *In-vivo* imaging

The distribution of renal targeting H-NPs in nude mice was investigated by imaging system (CRi Maestro, USA) (ex: 704 nm and filter: 735 nm). The ICG-H-NPs was injected via the tail vein (0.6 mg/mL). Blank group of animals received the same volume of saline. The mice were euthanasia 9 h after intravenous injection. The major organs including heart, liver, spleen and kidneys were collected for biodistribution analysis.

### Statistical Analysis

Statistical ananlysis was performed using Graphpad Prism 5 (Graphpad Softwear, La Jolla, CA). Image J was used for westernblot grayscale analysis. Student's *t* test (two-tailed) was used to compare experimental groups with controls. Wilcoxon signed-rank test was used to compare the expression of ENT1 between matched renal normal-tumor samples. Oneway-ANOVA analysis was used to test the difference between the means of several subgroups. *P* values < 0.05 were considered statistically significant. All statistical analyses were expressed as mean ± s.e.m. Each experiment was performed at least 3 independent replicates.

## Results

### Hypoxia-mediated inhibition of the demethylating effect of DAC

**Figure 1:**
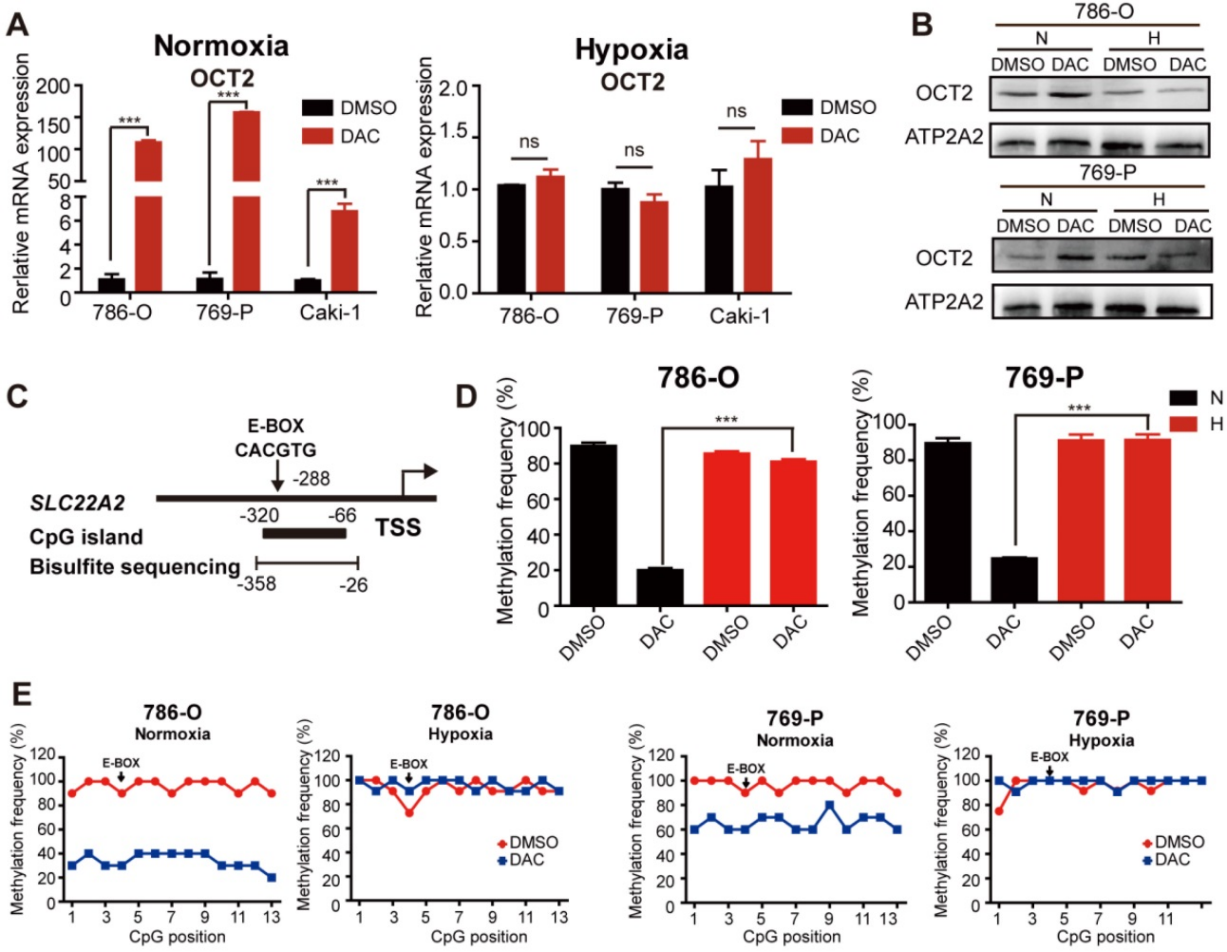
** The demethylation effect of DAC was blocked under hypoxia.** (A) Quantitative RT-PCR analysis of *SLC22A2* mRNA transcripts in 3 RCC cell lines (786-O, 769-P, Caki-1) after being treated with 2.5 μmol/L DAC for 72 h in normoxia and hypoxia. (B) Immunoblotting of OCT2 in 786-O and 769-P at 72 h after DAC treatment, membrane protein was extracted to determine the protein expression of OCT2. ATP2A2 was used as a loading control of membrane extracts. (C) Schematic diagram of CpG island, bisulfite sequencing region and E-BOX motif in the upstream of *SLC22A2* TSS. TSS, transcription start site. (D) MSRE-qPCR analysis of E-BOX methylation frequency in 786-O and 769-P cell lines, treated with DAC for 72 h in normoxia and hypoxia separately. (E) BSP analysis of each CpG site's methylation probability at the promoter of* SLC22A2* gene in 786-O and 769-P cell lines under normoxia or hypoxia, in the presence or absence of DAC for 72 h. The results of each group derives from at least 10 sequenced clones. Solid black dots indicate that the CpG site is methylated, while hollow dots indicate non-methylation. N, normoxia; H, hypoxia.

Given the hypoxic characteristic of RCC, we aimed to explore the effects of hypoxia on the demethylating activity of DAC in RCC cells. The mRNA and protein expression were detected in RCC cells after treatment with DAC for 72 h in normoxic (20% oxygen) and hypoxic (1% oxygen) conditions. Consistent with previous studies 8, both mRNA and protein expression of OCT2 was significantly upregulated by DAC under normoxia, whereas no significant transcriptional induction was observed in hypoxia (Figure 1A, B and Figure S1C). As shown in Figure 1C, a CpG island (CGI) containing 13 CpG sites was located within the OCT2 proximal promoter region, and the 4^th^ CpG site was identified inside the E-BOX motif (CACGTG). DNA methylation in this region was negatively correlated with the expression of OCT2. To address whether hypoxia contributed to DAC resistance in RCC cells through modulation of the demethylation effect of DAC, methylation-sensitive restriction enzyme-qPCR (MSRE-qPCR) analysis was used to detect the methylation frequency of the E-BOX motif. As shown in Figure 1D, after treatment with DAC, hypermethylation of the E-BOX motif was reduced in normoxic conditions. Conversely, in hypoxic conditions, DAC was unable to exert a DNA-demethylating function. To further evaluate the demethylating activity of DAC during hypoxia, bisulfite sequencing PCR (BSP) was applied to examine the methylation modifications of each CPG site after DAC treatment in normoxic and hypoxic conditions. The results were consistent with those of MSRE-qPCR analysis. In comparison with the dramatic decline in the probability of methylation under normoxia, hypermethylation was maintained at each CPG site after DAC treatment in hypoxic conditions (Figure 1E).

Previous research in our laboratory demonstrated that DNA hypermethylation coincided with a gene-specific loss of trimethylation at Lys4 of H3 (H3K4me3) at the OCT2 promoter. The chromatin immunoprecipitation (ChIP)-qPCR analysis was also performed to detect the abundance of H3K4me3 at various oxygen concentrations. Highly enriched H3K4me3 was observed around the TSS of OCT2 after DAC treatment under normoxia, whereas no significant upregulation was detected under hypoxia (Figure S1D). Together, hypermethylation and low H3K4me3 modification (a transcriptional repression signal) in the promoter region of OCT2 provided evidence that DAC was unable to upregulate the expression of OCT2 in hypoxic conditions.

### Hypoxia-mediated regulation of key factors in dynamic methylation of DNA

**Figure 2:**
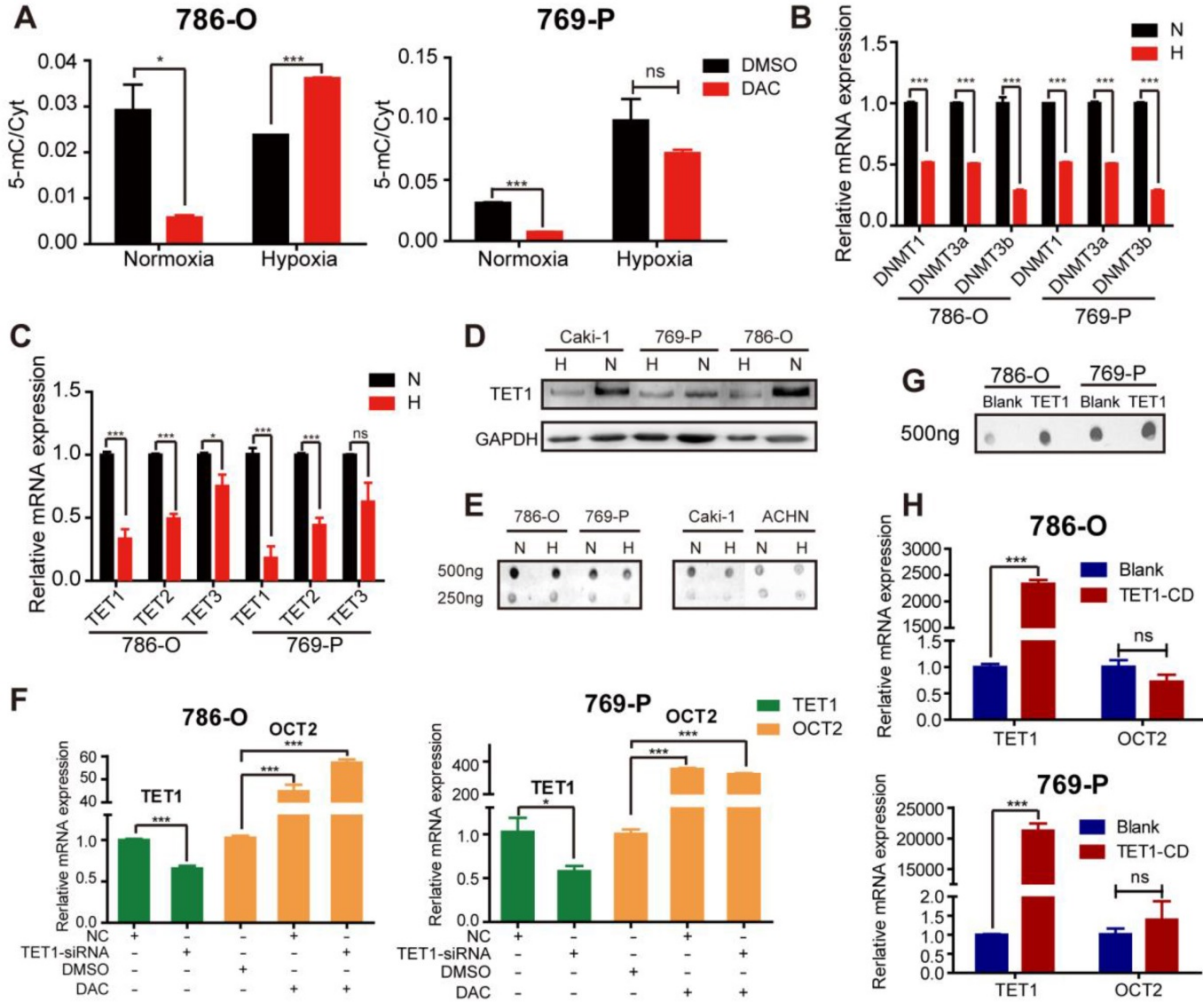
** Hypoxia mediated regulation of key factors in the process of DNA dynamic methylation.** (A) Proportion of 5-methylcytosine (5-mC) in total cytosine (Cyt) in 786-O and 769-P was detected after being treated with DAC for 72 h, in normoxia or hypoxia. The mRNA expression level of *DNMT1*, *DNMT3*, *DNMT3b* (B)and *TETs* (C)was significantly decreased during hypoxia in 786-O and 769-P compared with normoxia. Comparison of TET1 protein expression (D) and 5-hmC abundance (E) in 786-O, 769-P, Caki-1 and ACHN cell lines under hypoxia or normoxia by westernblot and dotblot separately. (F) The expression level of TET1 and OCT2 in 786-O and 769-P was detected by RT-PCR after TET1 knock-down and 72 h DAC treatment. (H) The expression level of *OCT2* were assayed in 786-O and 769-P cell lines transfected with TET1 catalytic domain expression vector or empty vector. The mRNA expression level of *TET1* (H) and the density of 5-hmC (G) were detected to ensure successful overexpression of TET1. TET1-CD, TET1 catalytic domain; N, normoxia; H, hypoxia.

As DAC is a broad-spectrum demethylating agent, to investigate whether the failure of demethylating activity was gene-specific, a liquid chromatography-mass spectrometry (LC-MS) method was employed to quantitatively detect cytosine (Cyt) and 5-methylcytosine (5-mC) content in RCC cell lines after treatment with DAC for 72 h in normoxic and hypoxic conditions. As displayed in Figure 2A, the amount of 5-mC as a proportion of total Cyt was remarkably decreased after DAC treatment under normoxia, whereas only a slight decrease was observed under hypoxia in 786-O RCC cells. Similar to *OCT2*, quantitative RT-PCR analysis of *OCT1*, *OCT3*, *OAT4*, and *PHT2*, which could be induced by DAC in normoxic conditions, revealed that the upregulation of these genes by DAC was decreased, or even disappeared, in hypoxic conditions (Figure S2A).

We further investigated the specific mechanisms of hypoxia-mediated DAC resistance in RCC cells by analyzing the expression of key factors in the process of dynamic DNA methylation, including DNMTs, TETs, AID, and TDG. Similar to the results of a previous study 18, hypoxia led to the downregulation of DNMTs, including *DNMT1*, *DNMT3a*, and *DNMT3b*, in RCC cells (Figure 2B). Given that hypoxia is linked to the decreased expression of DNMTs, no significant alteration was observed in matched human renal normal-tumor samples (Figure S2B); it is unlikely to be the predominant epigenetic change that prevents the demethylating activity of DAC. The activity of TETs (methylcytosine dioxygenases) was reported to be reduced by tumor hypoxia 19. The qPCR and immunoblotting analyses have demonstrated that the TET expression and the oxidizing activity of 5-mC to 5-hmC were suppressed by hypoxia in RCC cell lines (Figure 2C-E). A comparison of matched normal-tumor samples indicated that transcription, as well as the activity of TETs, were significantly decreased in RCC (Figure S2C, D).

Next, we attempted to investigate whether the hypoxia-mediated decrease in TET expression and activity overcame the demethylating function of DAC, leading to the accumulation of 5-mC and maintenance of the hypermethylated state. The silencing of TET1 was performed under normoxia to simulate the hypoxia-mediated TET1 repression, followed by DAC treatment for 72 h. We found that DAC still contributed to the transcriptional activation of OCT2 (Figure 2F). Overexpression of the TET1 catalytic domain did not alter the expression of OCT2 (Figure 2G, H), confirming that DAC resistance was independent of the hypoxia-associated changes in TET expression. Given the selectivity of the TET binding sites 25, we hypothesized that OCT2 was not the target gene of TET1. Besides, there was no significant change in the expression of TDG and AID in hypoxic conditions in RCC cells (Figure S2E), confirming that hypoxia did not interfere with the demethylation of OCT2 by DAC through regulating the dynamic methylation process.

### Hypoxia-mediated regulation of key factors in the DAC metabolic pathway

**Figure 3:**
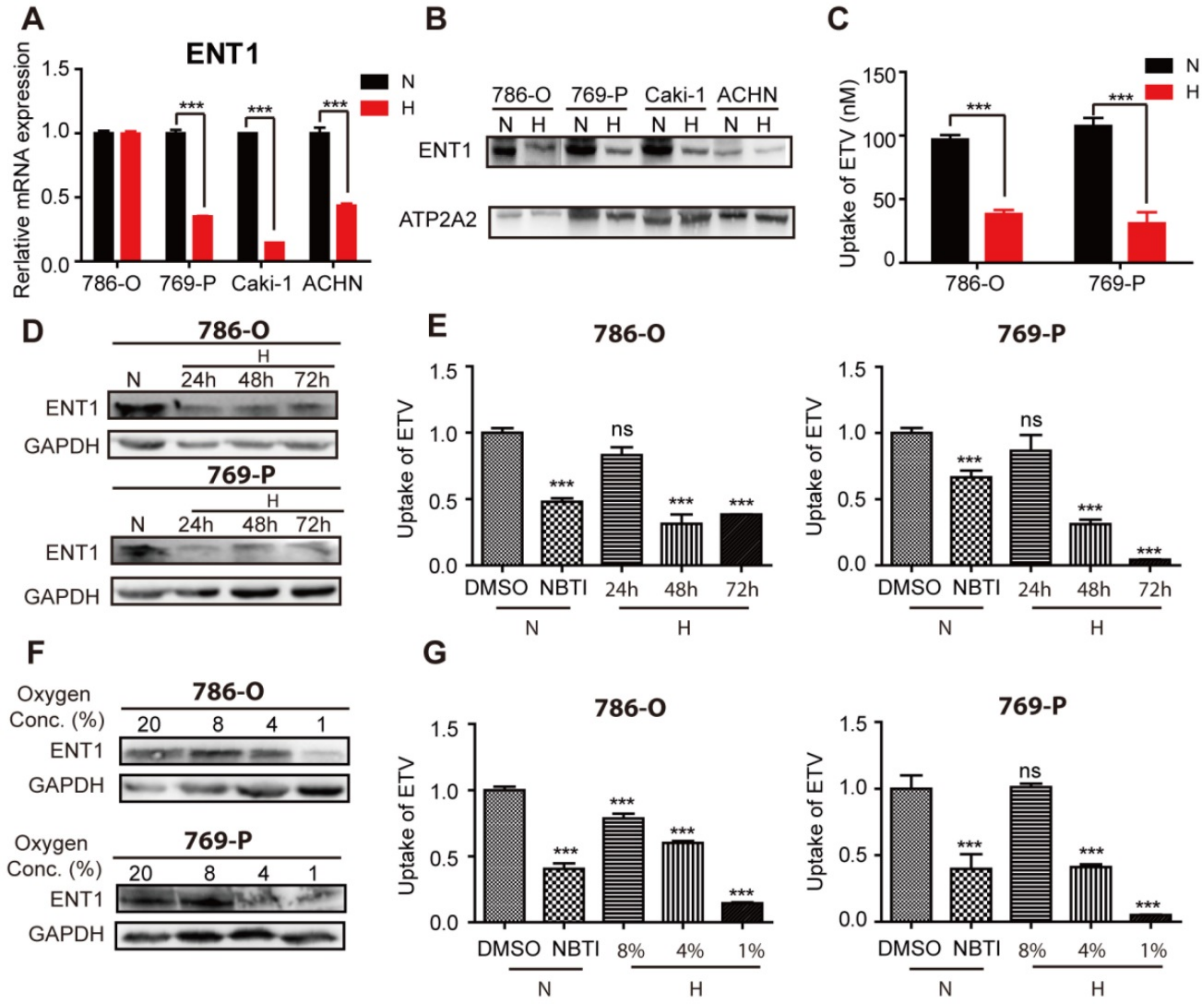
** Repression of ENT1 expression and uptake of substrate entecavir under hypoxia.** Four RCC cell lines (786-O, 769-P, Caki-1, ACHN) were exposed to normoxia or hypoxia for 48 h. Total RNA was isolated to determine *ENT1* mRNA levels by real-time PCR (A) and membrane protein was extracted to determine the protein expression of ENT1 (B). ATP2A2 was used as a loading control of membrane extracts. (C) Accumulation of entecavir, a substrates of ENT1, in 786-O and 769-P was detected by LC-MS after exposure under hypoxia for 48 h. The protein expression of ENT1 (D, F) and uptake of entecavir (E, G) were detected in 786-O and 769-P cell lines, exposed to indicated periods (24, 48 or 72 h) of hypoxia or indicated oxygen concentration (8%, 4% or 1%) for 48 h.

We attempted to identify the mechanisms that underpin OCT2 transcriptional repression by examining the transport and metabolism of DAC in hypoxic conditions. Consistent with a previous report 26, the mRNA expression profile of kidney tissues (n=3) indicated that concentrative nucleoside transporters (CNTs) were present at significantly lower levels than ENTs (Figure S3A). Further qPCR analysis (n=20) revealed that ENT1 was more abundant than ENT2 and CNT1 (Figure S3B). Also, the transcript of ENT1 was the most abundant among the five nucleoside transporters examined in RCC cells (Figure S3C), suggesting that DAC was mainly taken up by ENT1. Subsequently, we examined the influence of hypoxia on the expression of ENT1 in RCC cells. In addition to 786-O cells, the mRNA expression of ENT1 was significantly reduced in three other RCC cell lines (769-P, Caki-1-1and ACHN ) that were subjected to hypoxia for 48 h (Figure 3A). To extend these mRNA findings, we examined ENT1 protein expression in these four RCC cell lines. Notably, all four cell lines showed decreased ENT1 expression (Figure 3B), indicating the post-transcriptional regulation of ENT1 by hypoxia in RCC cells. The decreased accumulation of entecavir (ETV), a substrate of ENT1 27, further confirmed the hypoxia-associated repression of ENT1 expression in RCC cells (Figure 3C). Also, the effect of hypoxia was concentration- and time-dependent; ENT1 expression and activity of entecavir uptake showed repression at or below 4% oxygen for 48 h, or >48 h exposure in 1% oxygen (Figure 3D-G, S3D).

**Figure 4:**
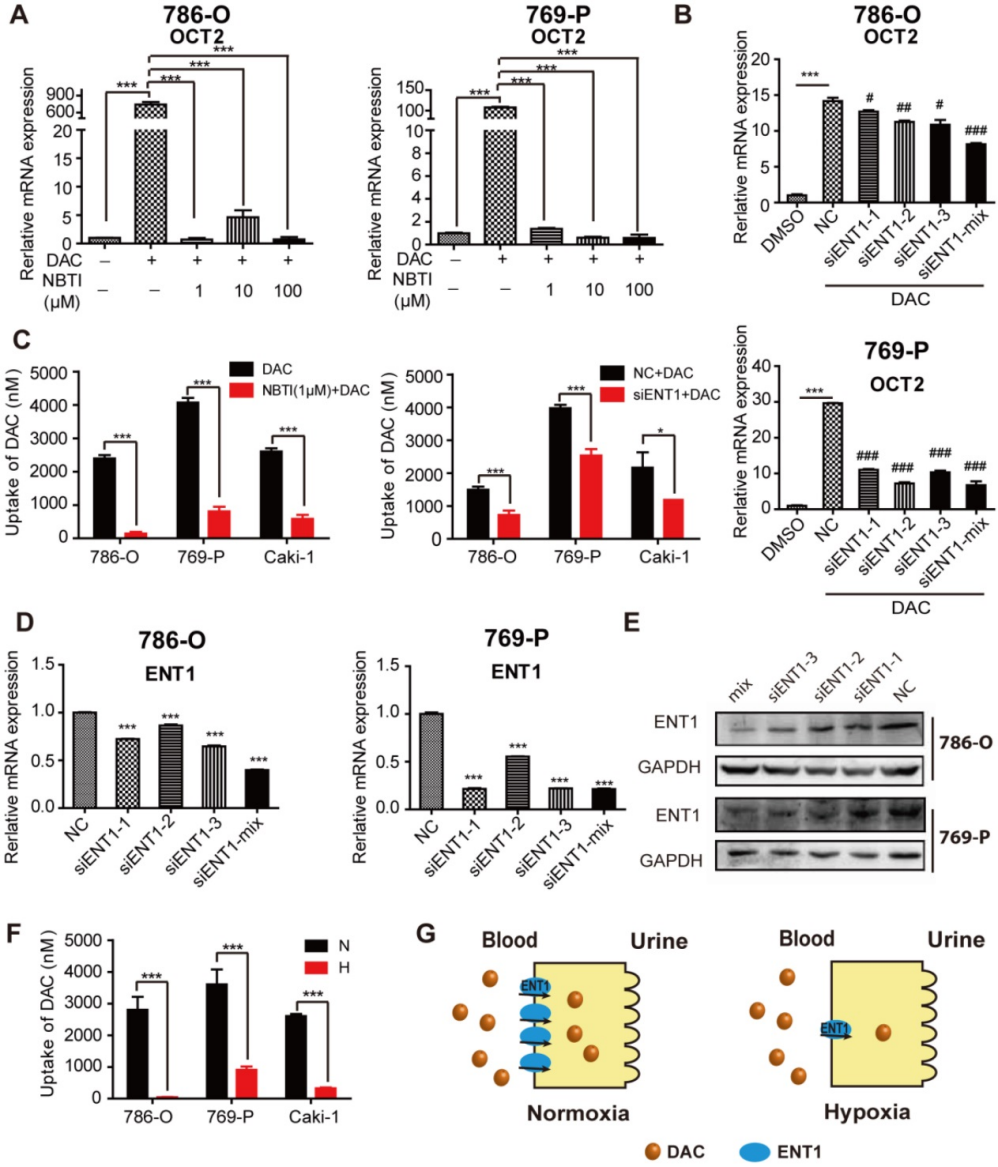
** Hypoxia-mediated reduction of DAC accumulation in RCC cells.** The mRNA expression level of *SLC22A2* was detected in 786-O and 769-P, treated with 2.5 μmol/L DAC for 72 h under normoxia , in the presence of 1, 10 , 100 μM NBTI (A) or after being transfected with 3 sequence specific ENT1 siRNAs separately or synchronously (B). (C) DAC accumulation was analyzed by LC-MS in 3 RCC cell lines (786-O, 769-P, Caki-1), incubated with 500 μM DAC at 37 °C for 3 min, in the presence of ENT1 inhibitor or pre-transfected with ENT1 specific siRNAs. The mRNA(D) and protein (E) expression level of ENT1 were deteced in 786-O and 769-P cell lines to verify siRNA knockdown inefficiency. (F) ENT1 uptake capacity of DAC was analyzed in 786-O, 769-P and Caki-1, exposed under hypoxia (1% oxygen concentration) for 48 h. (G) Schematic diagram of reduced DAC accumulation mediated by repressed ENT1 expression under hypoxia.

To assess whether the inhibition of ENT1 expression was one of the key determinants of DAC under hypoxia, a concentration gradient of S-(4-nitrobenzyl)-6-thioinosine (NBTI; NBMPR), an inhibitor of ENTs, which can inhibit ENT1 activity at low concentrations (0.1 μM) but inhibits ENT2 only at higher concentrations (100 μM), was used in conjunction with DAC administration. Analysis of *OCT2* transcript demonstrated that inhibition of ENT1 activity affected the induction of OCT2 transcriptional activation by DAC (Figure 4A), which was further confirmed by the knockdown of *ENT1* by short interfering RNA (siRNA) (Figure 4B, D, and E). Further, the accumulation of DAC was detected by using LC-MS to characterize the uptake of DAC by RCC cell lines. The time course of 500 μM DAC uptake and its concentration-dependence at 37 °C for 3 min in 786-O cells are shown in Figure S4A and B. The uptake of DAC was markedly decreased in cells treated with 1 μM NBTI or ENT1 siRNA, indicating that ENT1 was responsible for the uptake of DAC in RCC cells (Figure 4C). Conversely, virus packaging and stable cell selection were performed on 769-P cells to construct pCDH-ENT1, a cell line stably expressing ENT1, which exhibited increased uptake activity for both ETV and DAC, and stronger induction of *OCT2* by DAC compared with empty vector-transduced cells (Figure S4D-F). The loss of DAC accumulation was observed in hypoxia-triggered ENT1-suppressed RCC cells (Figure 4F).

Other than ENT1 expression, the activity of DAC was also affected by dCK and CDA 28. Therefore, we investigated whether they underpinned the DAC resistance in hypoxic conditions. No alteration in dCK expression was detected, and there was low expression of CDA in RCC cells (data not shown), which prevented abnormal DAC metabolism from causing hypoxia-associated DAC resistance.

### Repression of ENT1 in RCC

**Figure 5:**
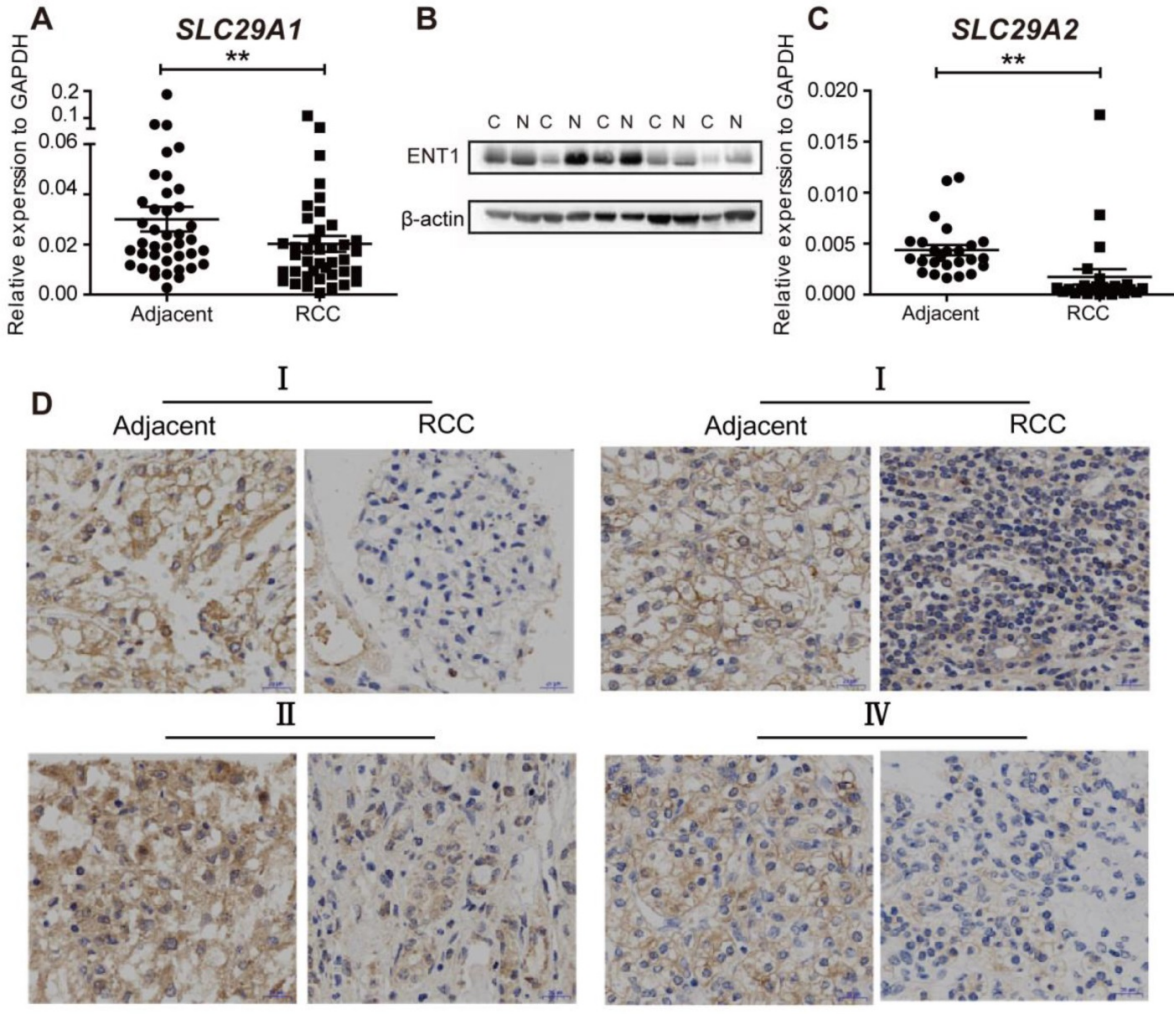
** The expresssion of ENT1 and ENT2 are downregulated in RCC.** (A, C) Comparison of *SLC29A1* (n=40) and *SLC29A2* (n=20) mRNA expression separately in human matched renal normal-tumor samples. The expression level was normalized to GAPDH. Results are expressed as mean ± SEM, Wilcoxon signed-rank test was used. **P<0.01. (B) Comparison of ENT1 protein expression in human matched renal normal-tumor samples (n=5). The blot of β-actin was probed as a control for protein loading. (D) Representative images of immunohistochemistry staining for ENT1 in adjacent non-tumor tissue and paired ccRCC tumor tissue at different TNM stages. Scale bar, 20 μm. The antibody 11337-1-AP (proteintech) against ENT1 was used in the immunohistochemistry assays.

We then probed whether RCC was resistant to DAC due to abnormal ENT1 expression. The expression of ENT1 (n=40) and ENT2 (n=20) was evaluated by using RT-PCR in human renal carcinoma samples and matched adjacent non-tumorous samples. As shown in Figure 5A and C, the expression of *SLC29A1* and *SLC29A2* was downregulated in RCC tissues, which was further confirmed by Western blotting (Figure 5B). Compared with ENT1, ENT2 showed a lower affinity for all nucleosides (except inosine) 29; downregulation of ENT2 expression in RCC led to further prevention of DAC uptake. Immunohistochemistry analysis of tumor and adjacent normal tissues at different stages of TNM revealed ENT1-negative staining in RCC tissues, indicating the widespread inhibition of ENT1 in the progression of RCC (Figure 4D).

### HIF-1α-mediated repression of ENT1 expression in Caki-1 cells under hypoxia

**Figure 6:**
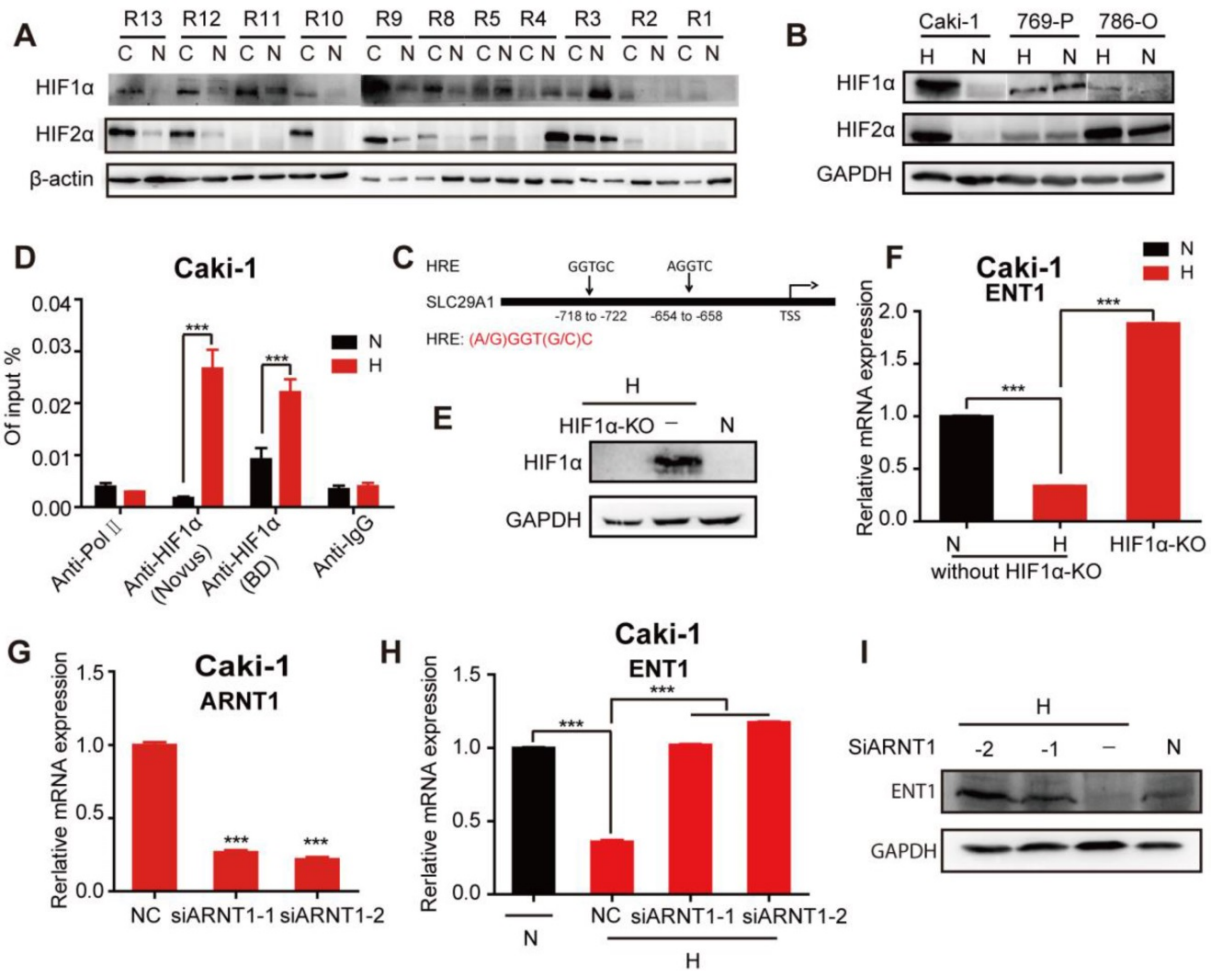
** HIF-1α-mediated repression of ENT1 expression in Caki-1 cells under hypoxia.** Comparison of HIF-1α and HIF-2α protein expression in human matched renal normal-tumor samples (n=11) (A) and 3 RCC cell lines (786-O, 769-P, Caki-1) under normoxia or hypoxia for 6 h (B). (C) Schematic diagram of two hypoxia response elements (HRE) in the promoter of ENT1. (D) CHIP-qPCR analysis of HIF-1α at the promoter of ENT1 in Caki-1 cells, exposed under normoxia or hypoxia for 48 h. Two different HIF-1α antibodies (610958, BD Biosciences; NB100-105, Novus) were used to pull down HIF-1α. The protein expression level of HIF-1α (E) and mRNA expression level of *SLC29A1* (F) in Caki-1 under normoxia or hypoxia and HIF-1α knockout-Caki-1 cells. (G) The expression level of ARNT1 (HIF-1β) was detected in Caki-1, transfected with ARNT1 specific siRNAs or NC. Student's *t* test (two-tailed) was used. (H, I) The expression level of ENT1 in Caki-1, transfected with ARNT1 specific siRNAs or NC, under normoxia or hypoxia. Oneway-ANOVA analysis was used. The results are expressed as mean ± SEM (n=3). ***P<0.001.

HIF-1α was reported to repress the expression of ENT1 30 and ENT2 31 in human epithelial cells. Exposure of different human tumor cells under hypoxia revealed a general downregulation of ENT1 expression (Figure S5A, B). The mechanism through which hypoxia regulated the expression of ENT1 was further explored due to its importance in the transport of nucleosides and their analogs. To determine whether HIF-1α participated in the regulation of ENT1, RCC cells were treated with a concentration gradient of DMOG (a prolyl hydroxylase inhibitor) in normoxia, which could stabilize nuclear HIF-1a protein expression and simulate the hypoxic microenvironment. Like the phenotype in hypoxic conditions, RCC cells exhibited a DAC-resistant phenotype after DMOG treatment, comprising decreased ENT1 expression, reduced DAC and ETV uptake, and suppressed induction of OCT2 by DAC (Figure S6A-D). Stable expression of HIF-1α and HIF-2α was detected in most RCC tissues in 11 pairs of matched renal normal-tumor samples, but was only observed in Caki-1 cells (Figure 6A, B); therefore, these cells were used for further mechanistic studies.

The ChIP assay performed on Caki-1 cells revealed a higher abundance of HIF-1α occupancy around the hypoxia-responsive element (HRE) in the ENT1 promoter under hypoxia (Figure 6C, D). The HIF-1a loss or gain of function illustrated the central role of HIF-1a in the transcriptional repression of ENT2 in Caki-1 cells (Figure S6E, F). We also showed that the inactivation of HIF-1α by using CRISPR/Cas9 under hypoxia successfully reversed hypoxia-associated ENT1 inhibition (Figure 6E, F). It has been reported that stabilized HIF-α translocates into the nucleus and dimerizes with HIF-1β/ARNT 15. Similarly, the downregulation of ARNT by two highly effective siRNAs resulted in the re-expression of ENT1 in hypoxia (Figure 6G-I). These results strongly suggested that the transcriptional repression of ENT1 in Caki-1 cells was caused by the stabilized expression of HIF-1a under hypoxia.

### Effects of re-oxygenation on hypoxia-induced DAC resistance

**Figure 7:**
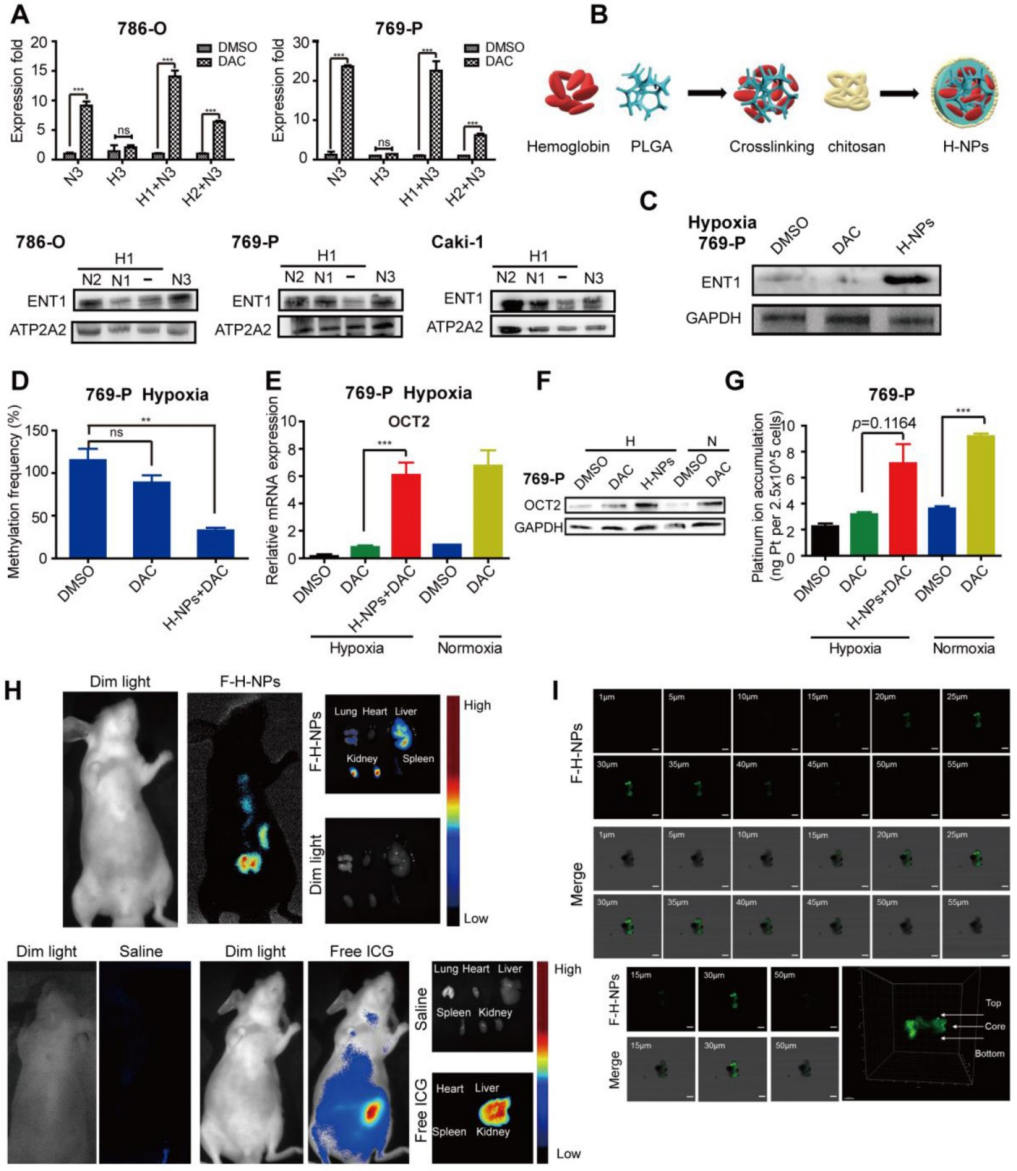
** Effects of re-oxygenation on hypoxia-induced DAC resistance.** (A) Quantitative RT-PCR analysis of *SLC22A2* mRNA in 786-O and 769-P, treated with 2.5μmol/L DAC for 72h in normoxia, after exposed under hypoxia for 24 or 48 h and The protein expression level of ENT1 in 786-O, 769-P and Caki-1, exposed under normoxia for indicated time, after exposed under hypoxia for 24 h. (B) Schematic of Hemoglobin-based nanocarrier preparation and structure. (C) Western blotting of ENT1 treated by H-NPs in 769-P under hypoxia. (D) MSRE-qPCR analysis of E-BOX methylation frequency in 769-P cell lines, treated with DAC, in the presence or absence of H-NPs, for 72 h in hypoxia separately. The mRNA (E) and protein (F) level expression of OCT2 and oxaliplatin uptake (G) in 769-P cell lines, treated with DAC, in the presence or absence of H-NPs, for 72 h in hypoxia separately. (H) Representative *in-vivo* and *ex-vivo* fluorescence images of nude mice treated with saline, Free ICG and ICG-H-NPs 5 h post injection. (I) The z-stacking images of the 769-P spheroid at different sections from 1 to 55 μm, sections from 20-40 μm had high fluorescence intensity and confocal microscope images of spheroid core and 3D graphics constructed by Imaris 9.3.1. The high fluorescence intensity at the core of 769-P spheroid which arrowed in 3D-plot indicated the H-NPs could alleviate hypoxic core of the solid tumor *in vivo*.

Finally, we assessed whether re-oxygenation could reverse DAC resistance under hypoxia. RCC cells pre-cultured under hypoxia for 1 or 2 days were transferred to normoxic conditions and then incubated with DAC for an additional 3 days. RT-PCR analysis revealed that, despite the prolonged period of hypoxia, DAC was still able to activate OCT2 expression in RCC cells after transfer to normoxia. Re-expression of the repressed ENT1 further suggested that re-oxygenation was a viable method to overcome DAC resistance under hypoxia (Figure 7A).

The hemoglobin-based oxygen nanocarriers H-NPs were constructed, with poly (lactic-co-glycolic acid) (PLGA) to load hemoglobin by hydrophobic interaction, and coated with a low molecular weight hydroxyethyl chitosan to target the kidney (Figure 7B). H-NPs were shown to have a homogenous dimension with small particle size, moderate stability in the aqueous phase, and efficient oxygen-carrying ability (Figure S7). To monitor the cellular uptake of H-NPs, we developed a dual-fluorescence-stained H-NPs, F-D-H-NPs, which contained FITC-stained chitosan and Dil-stained PLGA. The cellular accumulation of H-NPs was measured with a confocal fluorescence imaging system (Figure S8A). The restoration of ENT1 expression in hypoxia after treatment with H-NPs indicated that the oxygen nanocarrier was able to alleviate the hypoxia-mediated repression phenotype of ENT1 (Figure 7C). We then tested the role of H-NPs in DAC demethylation and detected a remarkable decrease in methylation frequency at the E-BOX motif (Figure 7D), upregulated OCT2 expression (Figure 7E, F), and increased oxaliplatin accumulation (Figure 7G). These observations demonstrated that H-NPs could attenuate the hypoxia-induced loss of DAC activity and sensitize RCC cells to the sequential combination therapy of DAC and oxaliplatin.

To further investigate the potential clinical application of H-NPs, renal targeting and their ability to penetrate solid tumor spheroid core were detected. ICG was used as a fluorescent label to prepare ICG-H-NPs for investigating their *in vivo* targeting ability. The size distribution and fluorescence spectrum of ICG-H-NPs indicated that the accumulation of H-NPs could be tracked *in vivo* (Figure S8B, C). Compared with the group treated with free ICG, which targeted the liver, fluorescence was observed clearly in the renal region of nude mice at 5 h after ICG-H-NPs injection, which indicated effective renal targeting (Figure 7H). A three-dimensional RCC cell culture model was used to investigate the core penetration ability of H-NPs under hypoxia. Z-stacking images from 1 to 55 μm depth of 769-P spheroid cell cultures showed significantly high fluorescence in the core of spheroids (Figure 7I), indicating that H-NPs permeated deeply into the tumor matrix and resulted in a high uptake in the hypoxic RCC cell spheroid model.

### Hypoxia-mediated modification of histone acetylation for the regulation of OCT2 expression

**Figure 8:**
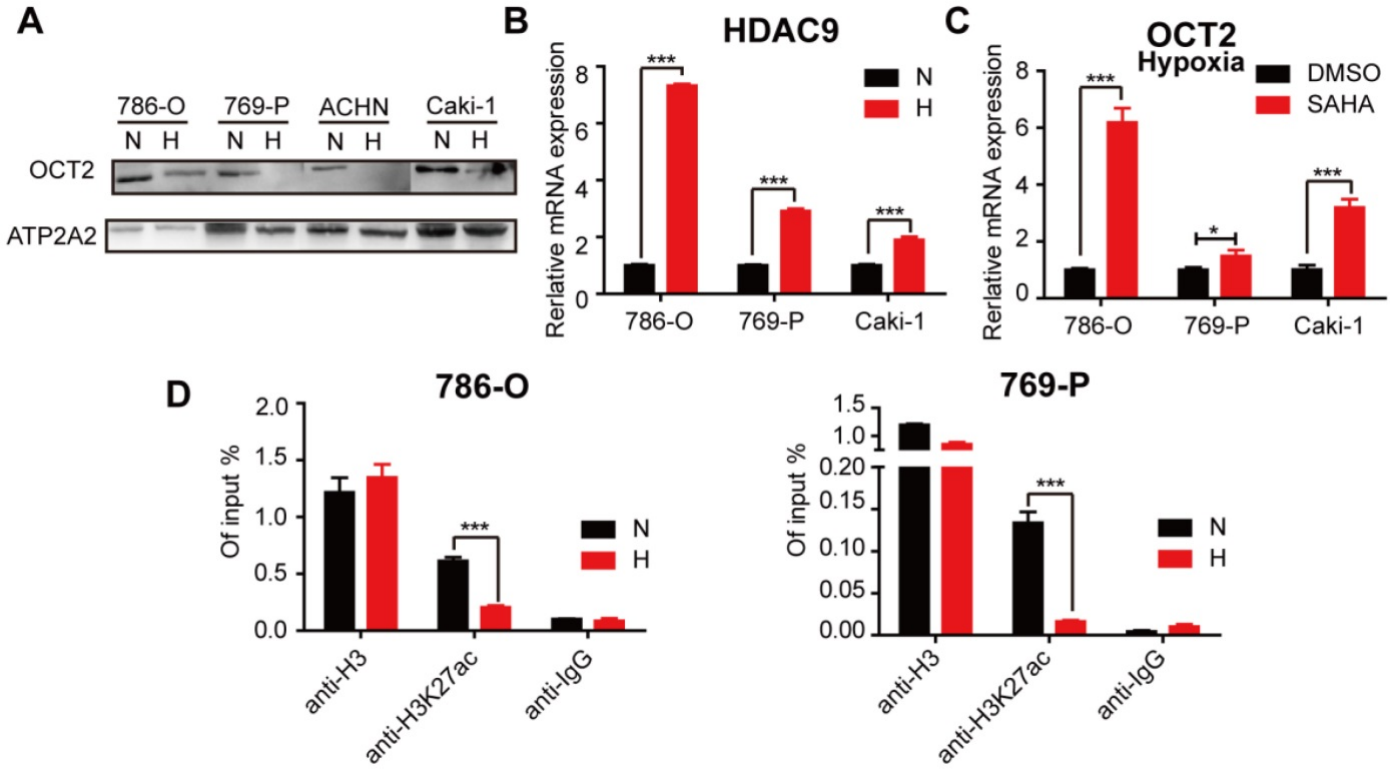
** Hypoxia-mediated modification of histone acetylation for the regulation of OCT2 expression.** (A) Four RCC cell lines (786-O, 769-P, Caki-1 and ACHN) were exposed to normoxia or hypoxia for 48 h. Membrane protein was extracted to determine the protein expression of OCT2. ATP2A2 was used as a loading control of membrane extracts. N, normoxia; H, hypoxia. (B) Quantitative RT-PCR analysis of *HDAC9* mRNA expression level in 3 RCC cell lines (786-O, 769-P, Caki-1), exposed under normoxia or hypoxia for 48 h. (C) Quantitative RT-PCR analysis of *SLC22A2* mRNA transcript in 3 RCC cell lines (786-O, 769-P, Caki-1) after being treated with 2 μmol/L SAHA for 48 h in hypoxia. (D) The enrichment of H3K27ac modification level at the OCT2 promoter region in 786-O and 769-P was detected by CHIP-qPCR analysis after exposed to normoxia or hypoxia for 48 h.

Previously, we found that histone acetylation was involved in the regulation of OCT2 in RCC 32. The protein expression of OCT2 was decreased in hypoxic conditions (Figure 8A), suggesting that hypoxia contributed to OCT2 repression in RCC. We then explored whether hypoxia triggered the alteration of histone modifications in the OCT2 promoter. As shown in Figure 8B, the expression of histone deacetylase 9 (HDAC9) was upregulated, and SAHA, a histone deacetylase inhibitor, activated the expression of OCT2 under hypoxia (Figure 8B, C). No changes in HDAC7 expression were detected (data not shown). Given the widespread decrease in H3K27ac (acetylation) in RCC cells in hypoxic conditions (Figure S9A, B), the ChIP assay was performed to detect the abundance of H3K27ac in the OCT2 promoter (Figure 8D and Figure S9D). The results indicated that upregulation of histone deacetylase activity of HDAC9 driven by hypoxia underpinned the dramatic decrease in H3K27ac modification at the OCT2 promoter that led to the transcriptional inhibition of OCT2.

## Discussion

Emerging evidence has indicated that the expression of glyceraldehyde-3-phosphate dehydrogenase (GAPDH), considered to be a housekeeping gene and often used as an internal control, is deregulated in various cancers and can be modulated by several cancer-related factors 33. Hypoxia is reported to stimulate GAPDH gene expression in numerous cells 34-36. We compared the changes in GAPDH and β-actin expression both at mRNA and protein levels in hypoxic conditions and found that the protein expression of both genes was essentially unchanged. However, at the mRNA level, the changes in GAPDH were smaller than those in β-actin (Figure S1A, B), suggesting that GAPDH might be a more suitable internal control in RCC cells.

TET1 is involved in tumor inhibition by promoting cell apoptosis and inhibiting cell proliferation and invasion 37. Recent reports indicated that there was a markedly low expression of TET1 in RCC 37 and the loss of 5-hmC, a hallmark of ccRCC, was linked to hypermethylation in tumors and associated with shorter overall survival and poor prognosis 38. Our results showed that tumor hypoxia directly reduced the expression and activity of TET, causing a global decrease in 5-hmC in RCC cells. Although TET1 did not exert a demethylation effect on OCT2, 5-hmC loss in kidney cancer has been reported to be associated with the downregulation of IDH1^38^ and VHL^39^. As hypoxia is pervasive in tumors, a hypoxic microenvironment was speculated to be one of the mechanisms that triggered the reduced expression of TETs and drove hypermethylation in solid tumors. Further investigations are needed to elucidate the mechanisms of the hypoxia-mediated downregulation of TETs and explore methods of 5-hmC restoration in RCC.

ENT1, which is almost ubiquitously distributed in human tissues, is critical for the regulation of endogenous nucleosides (e.g., adenosine), and the uptake of chemotherapeutic nucleoside analogs, including gemcitabine, didanosine, cytarabine, and ribavirin 40. Studies have shown that ENT1 transport can be regulated by protein kinase C (PKC) 41, inflammatory cytokines 42, and the JNK-cJun pathway 43. Also, the tumor suppressor F-box and WD repeat domain-containing 7 (FBW7) proteins can regulate ENT1 expression at a post-transcriptional level, whereas lysosomal inhibition can increase the protein expression of ENT1 44. In the present study, we also investigated the regulation of ENT1 expression by miRNAs at the post-transcriptional level. Several miRNAs, such as miR-185-5p, miR-124-3p, miR-210-3p, miR-122-5p, miR-214-5p, miR-223-3p, miR-34b-5p, and miR-506-3p, which were predicted to target ENT1 and reported to be upregulated in RCC, did not downregulate the transport activity of ENT1 (data not shown). However, the lysosome inhibitor chloroquine (CQ) induced ENT1 expression in hypoxic conditions (Figure S10A), which was consistent with a previous report 44. As FBW7 expression was found to be reduced under hypoxia (Figure S10B), our findings also suggested potential new avenues of investigation into FBW7-related mechanisms of the hypoxia-mediated regulation of ENT1.

In this study, DMOG-treated RCC cells under normoxia displayed a DAC-resistant phenotype. However, consistent with a previous report, the protein expression of HIF-1α was not detectable in 786-O and 769-P cells in hypoxic conditions 45. DMOG can competitively inhibit α-KG-dependent dioxygenases, including histone demethylases and TET 5-metyhlcytosine hydroxylases. The inhibition of histone 3 lysine 27 demethylase KDM6A (UTX) could downregulate ENT1 expression through a reduction in H3K27 acetylation at the ENT1 locus 46. We, therefore, speculated that DMOG decreased the expression of ENT1 through the regulation of histone demethylase activity. The mechanisms by which DMOG participates in the regulation of ENT1 and the association between ENT1 expression and histone modification require further investigation.

The resistance of tumor cells to anticancer drugs is generally caused by impaired drug delivery or genetic and epigenetic alterations that affect drug sensitivity 47. We demonstrated that hypoxia blocked DAC delivery by repressing ENT1 expression, which led to DAC resistance in RCC cells. It is generally believed that hypoxia can induce therapeutic resistance by activating the expression of drug efflux transporters (such as MDR1, MRP1, and BCRP), metabolic reprogramming, inhibiting apoptosis and senescence, and reducing DNA damage 15. The upregulation of P-gP in RCC tumor tissues has also been reported 48. Also, MATE2K, an efflux transporter, which is expressed on the apical membrane of proximal tubules and mediates the excretion of oxaliplatin from the kidney, has been shown to be dramatically repressed in RCC 49, 50. Whether hypoxia participates in the regulation of MATE2K expression remains to be further studied. Further research into the mechanisms of hypoxia-mediated drug resistance is imperative to circumvent drug resistance and improve chemotherapy.

The current strategies for overcoming the hypoxic microenvironment of tumors include target/ leverage hypoxia by obligate anaerobe or hypoxia-activated prodrugs, specific targeting of HIFs or UPR and mTOR pathways important in hypoxic cells, and re-oxygenation or vascular normalization by oxygen nanocarriers 51, 52. In this study, we found that re-oxygenation was a viable method to overcome DAC resistance under hypoxia. However, YC-1 and PT-2385, specific inhibitors of HIF-1α and HIF-2α, could neither upregulate the expression of ENT1 nor reverse DAC resistance under hypoxia (data not shown). Therefore, the development of an exogenous oxygen carrier with renal targeting function and biosafety features is expected to alleviate hypoxia in RCC and might be important for the application of epigenetic drugs against the resistance of RCC to chemotherapeutics. Hemoglobin was used in our model as a biosafe oxygen carrier. As an iron-containing oxygen-transport metalloprotein, each hemoglobin was able to carry four oxygen molecules, with an oxygen-binding capacity of 1.34 mL O_2_ per gram 53.

Oxygen concentration in normal kidney tissue has been reported to be 9.5% 21. As shown in Figure S11, under 9.5% oxygen concentration, the ability of DAC to induce SLC22A2 expression was consistent with 20% oxygen. Furthermore, we found that DAC could not induce OCT2 under 4% oxygen concentration. Considering that H-NPs could relieve hypoxia but not as well as normoxia, we speculated that H-NPs could increase the oxygen content of RCC to 4%~9.5%. In our study, the outer surface of H-NPs was chitosan, which has been proved to have renal targetability 53. Also, the particle size of H-NPs was about 130 nm, indicating that it could preferentially target the tumor by enhanced permeability and retention (EPR) effect 54. The *in-vitro* antitumor efficiency study indicated that this treatment strategy could slightly reverse oxaliplatin resistance of RCC cells under hypoxia (Figure S8D). In the future, the antitumor efficiency of this treatment strategy will be studied *in vivo,* and folate-fusion H-NPs will be constructed to optimize the nanocarrier and improve its targeting to tumor sites.

## Conclusions

Collectively, our results showed that the hypoxia-mediated repression of ENT1, which was also exceptionally suppressed in RCC, led to the inability of DAC to upregulate the expression of OCT2 in hypoxic conditions. The hypoxia-induced upregulation of HDAC9 decreased H3K27ac modification at the OCT2 promoter, leading to the transcriptional inhibition of OCT2. A simple and efficient oxygen nanocarrier based on hemoglobin was developed to alleviate resistance to oxaliplatin and DAC in RCC cells under hypoxia, which may have potential clinical applications.

## Supplementary Material

Supplementary figures and tables.

## Abbreviations

RCC: renal cell carcinoma
mRCC: metastatic renal cell carcinoma
ccRCC: clear cell renal cell carcinoma
DAC: decitabine
hENT1: human equilibrative nucleoside transporter 1
TSS: transcriptional start sites
DNMTs: DNA methyltransferases
5-mC: C-5 position of cytosine
5-hmC: 5-hydroxymethylcytosine
TET1: ten-eleven-translocation 1
AID: activation-induced deaminase
HIF: hypoxia-inducible factors
MSRE-qPCR: methylation-sensitive restriction enzymes-qPCR
BSP: bisulfite sequencing PCR
CGI: CpG island
H3K4me3: trimethylation at Lys4 of H3
HDAC9: histone deacetylase 9
H3K27ac: acetylation on lysine 27 of histone 3
LC-MS: liquid chromatography-mass spectrometry
ChIP: Chromatin Immunoprecipitation
OCT2: Organic cation transporter 2
E-BOX: Enhancer box
H-NPs: hemoglobin-based oxygen nanocarrier
ETV: entecavir
NBTI: S-(4-nitrobenzyl)-6-thioinosine
