# Supplementary material for: The failure of DAC to induce OCT2 expression and its remission by hemoglobin-based nanocarriers under hypoxia in renal cell carcinoma
Source: Theranostics. 2020 Feb 18;10(8):3562–78. doi: 10.7150/thno.39944 (PMC7069078; doi:10.7150/thno.39944)
Supplement: Supplementary file 1 — Supplementary figures and tables. [file thnov10p3562s1.pdf]

Appendix A. Supporting information

List of Supplementary Materials

Supplementary figures

Fig. S1 The demethylation effect of DAC was blocked under hypoxia.

Fig. S2 Hypoxia mediated regulation of key factors in the process of DNA dynamic methylation.

Fig. S3 Repression of ENT1 expression under hypoxia.

Fig. S4 The interaction of DAC with ENT1.

Fig. S5 Repression of ENT1 mRNA, protein expression under hypoxia is ubiquitous in different cancer cells.

Fig. S6 DMOG treatment-mediated DAC resistance in RCC cell lines under normoxia.

Figure.S7 Characterizations of H-Nps.

Figure.S8 The cellar uptake and antitumor efficiency study of H-Nps.

Figure.S9 Hypoxia-mediated modification of histone acetylation for the regulation of OCT2 expression.

Figure.S10 FBW7-mediated regulation of ENT1 under hypoxia.

Figure.S11 Induction of OCT2 by DAC under normal renal oxygen concentration.

Supplementary tables

Table S1. Primers used in this study

Table S2. SiRNAs used in this study

Table S3. Tissue specimen information

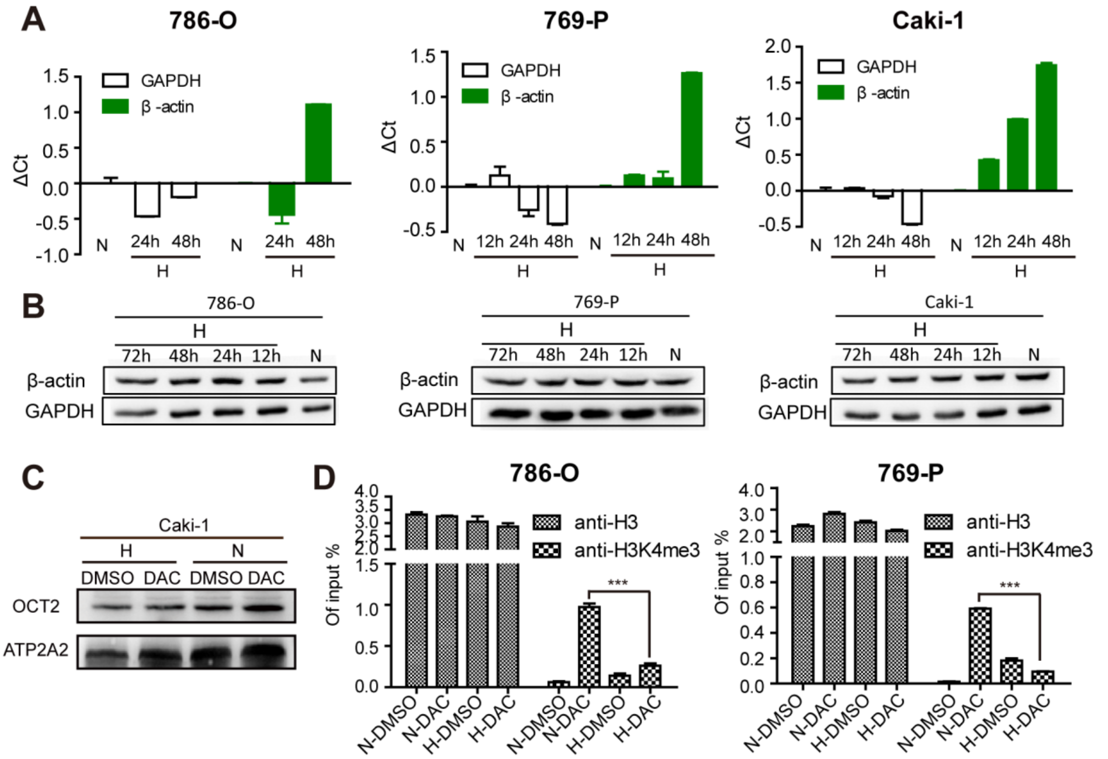

Figure.S1 The demethylation effect of DAC was blocked under hypoxia. (A, B) The mRNA and protein expression level of *GAPDH* and *β-actin* in 3 RCC cell lines (786-O, 769-P, Caki-1) were detected under normoxia and hypoxia. ΔCt means $Ct_{hypoxia}$- $Ct_{normoxia}$. (C) Immunobotting of OCT2 in Caki-1 at 72 h after DAC treatment, membrane protein was extracted to determine the protein expression of OCT2. ATP2A2 was used as a loading control of membrane extracts. (D) CHIP-qPCR analysis of H3K4me3 modification at *SLC22A2* promoter in 786-O and 769-P cells, treated with DAC for 72h, in normoxia or hypoxia. N, normoxia; H, hypoxia. Student's *t* test (two-tailed) was used. The results are expressed as mean ± SEM (n=3). ****P*<0.001.

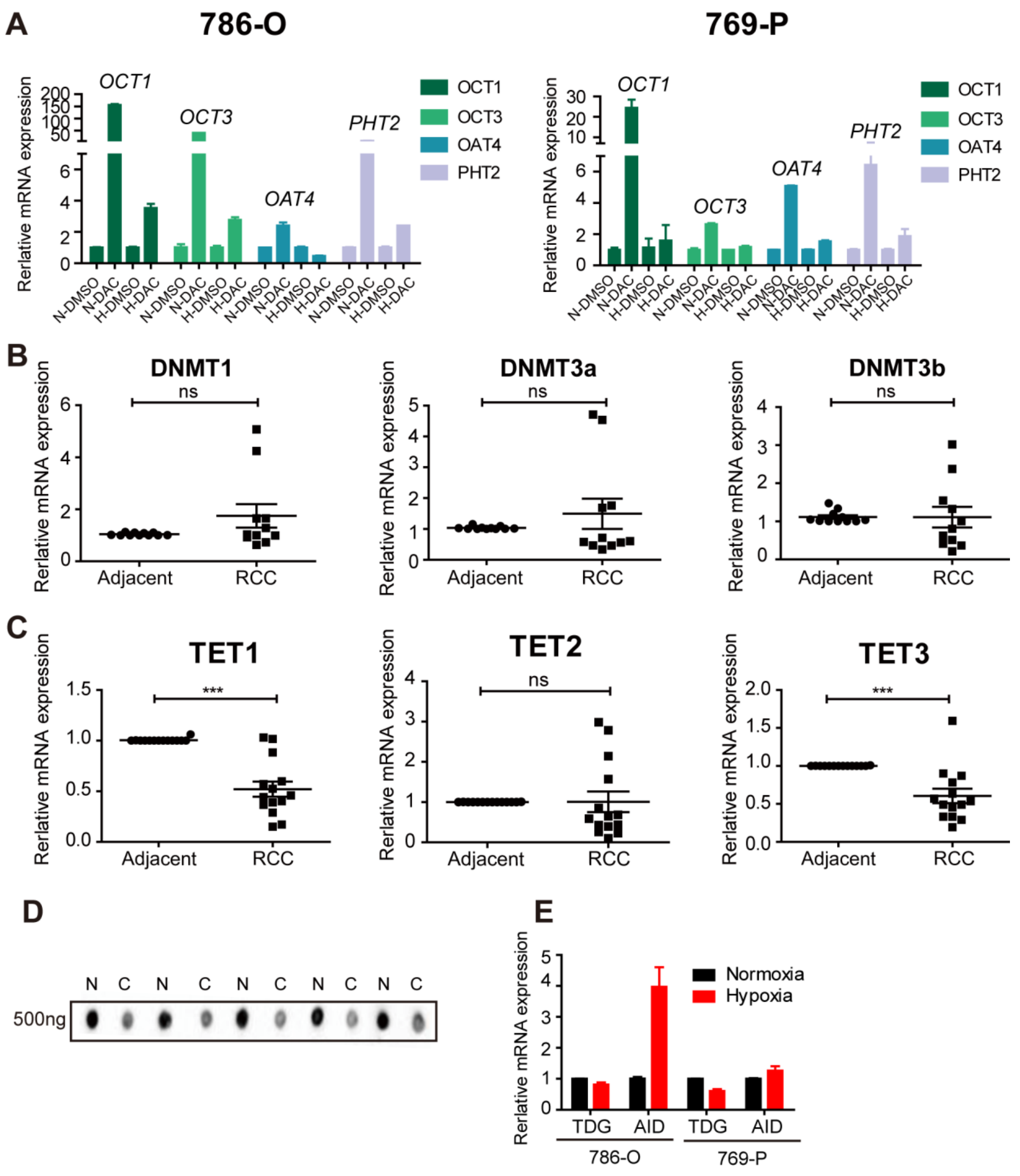

Figure.S2 Hypoxia mediated regulation of key factors in the process of DNA dynamic methylation. (A) Quantitative RT-PCR analysis of *OCT1*, *OCT3*, *OAT4* and *PHT2* mRNA transcripts in 786-O and 769-P after being treated with 2.5 μmol/L DAC for 72 h in normoxia and hypoxia. (B, C) The mRNA expression level of *DNMTs* (n = 11) and *TETs* (n = 15) in RCC tissues and paired tumor-adjacent tissues was detected by RT-PCR. The expression level was normalized to *GAPDH*. Results are expressed as mean ± SD, Wilcoxon signed-rank test was used. ****P*<0.001. (D) Dot Blot analysis was used to detect the distribution of 5-hmC in RCC tissues and matched normal tissues (n = 5). N, normal; C, cancer. (E) The expression of *TDG* and *AID* in 786-O and 769-P cells under hypoxia and normoxia was detected by RT-PCR.

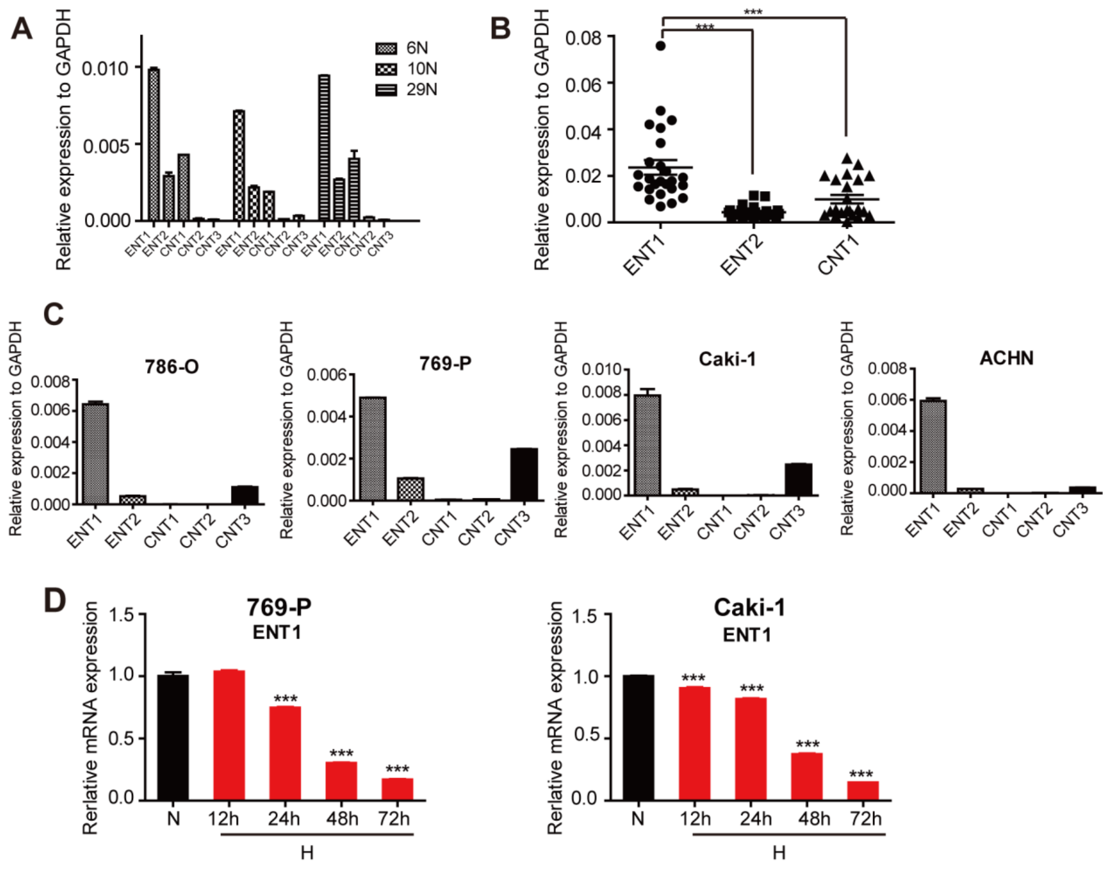

Figure.S3 Repression of ENT1 expression under hypoxia. (A) Quantitative RT-PCR analysis of the mRNA expression level of *SLC29A1*, *SLC29A2* and *SLC28A1*, *SLC28A2*, *SLC28A3* in kidney tissue. Results are expressed as mean ± SEM (n=3). (B) The mRNA expression level of *SLC29A1*, *SLC29A2* and *SLC28A1* in kidney tissue. The expression level was normalized to GAPDH. Results are expressed as mean ± SEM (n=20). (C) Quantitative RT-PCR analysis was used to detect the abundance of *SLC29A1*, *SLC29A2* and *SLC28A1*, *SLC28A2*, *SLC28A3* in four RCC cell lines (786-O, 769-P, Caki-1, ACHN). The expression level was normalized to GAPDH. (D) 769-P and Caki-1 were exposed to normoxia (72 h) or hypoxia (12, 24, 48 or 72 h) to detect the hypoxia exposure time-dependent repression of *ENT1* mRNA expresssion.

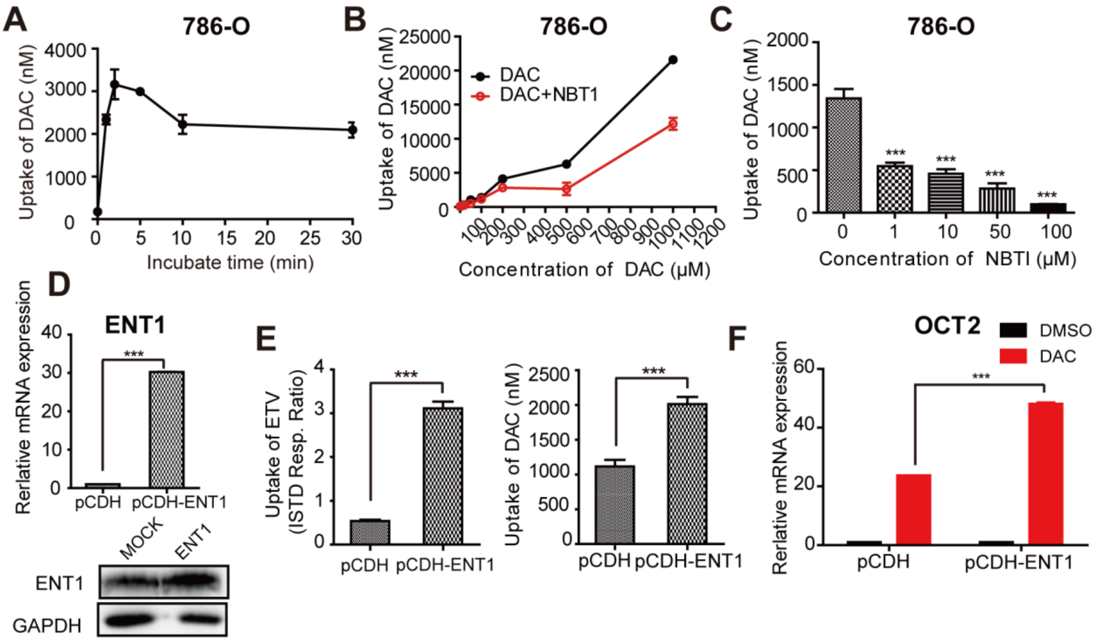

Figure.S4 The interaction of DAC with ENT1. Time course and concentration dependency of DAC uptake. (A) DAC accumulation in 786-O at 1, 3, 5, 10,30 min after incubation at concentration up to 500 μM. (B) Concentration-dependent profiles of DAC in 786-O with or without 1μM NBTI. (C) Uptake of DAC in 786-O cells , incubated with 500 μM DAC and designated concentration of NBTI at 37°C for 3 min. ENT1 was overexpressed in 769-P by lentivirus infection. The mRNA, protein expression level of ENT1 (D) and uptake of ETV and DAC (E) were detected in mock cell lines (pCDH) and ENT1 overexpressing cell lines (pCDH-ENT). (F) Up-regulation of *SLC22A2* by DAC was detected by qRT-PCR in ENT1 overexpressing cell lines.Results are expressed as mean ± SEM (n = 3). Student's *t* test (two-tailed) was used. The results are expressed as mean ± SEM (n=3). ****P*<0.001.

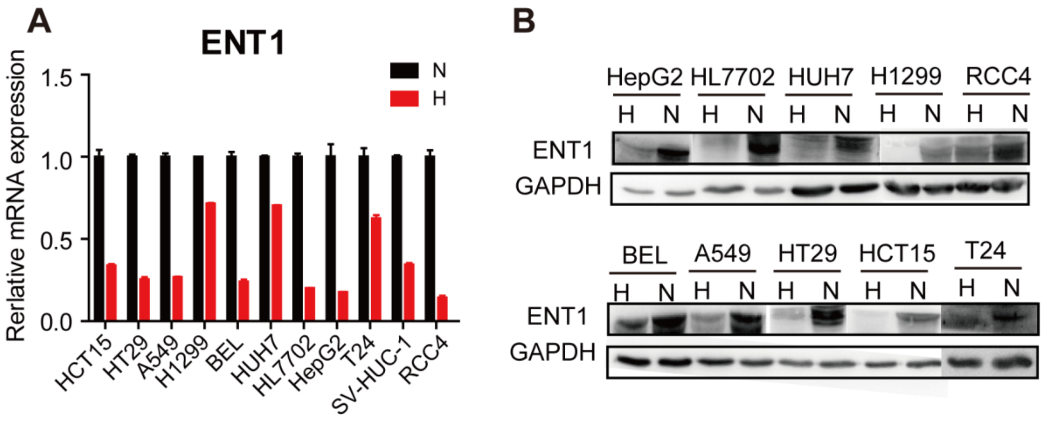

Figure.S5 Repression of ENT1 mRNA, protein expression under hypoxia is ubiquitous

in different cancer cells. The mRNA and protein expression level of ENT1 was detected in human colorectal cancer cell lines (HCT-15, HT29), non-small cell lung cancer cell lines (A549, H1299), hepatoma cell lines (Bel-7402, Huh7, HEPG2), human liver HL-7702 cells, bladder cancer cell lines (T24, SV-HUC-1) and clear cell renal cell line carcinoma RCC-4, exposed under normoxia or hypoxia for 48 h, separately. Results are expressed as mean ± SEM (n = 3).

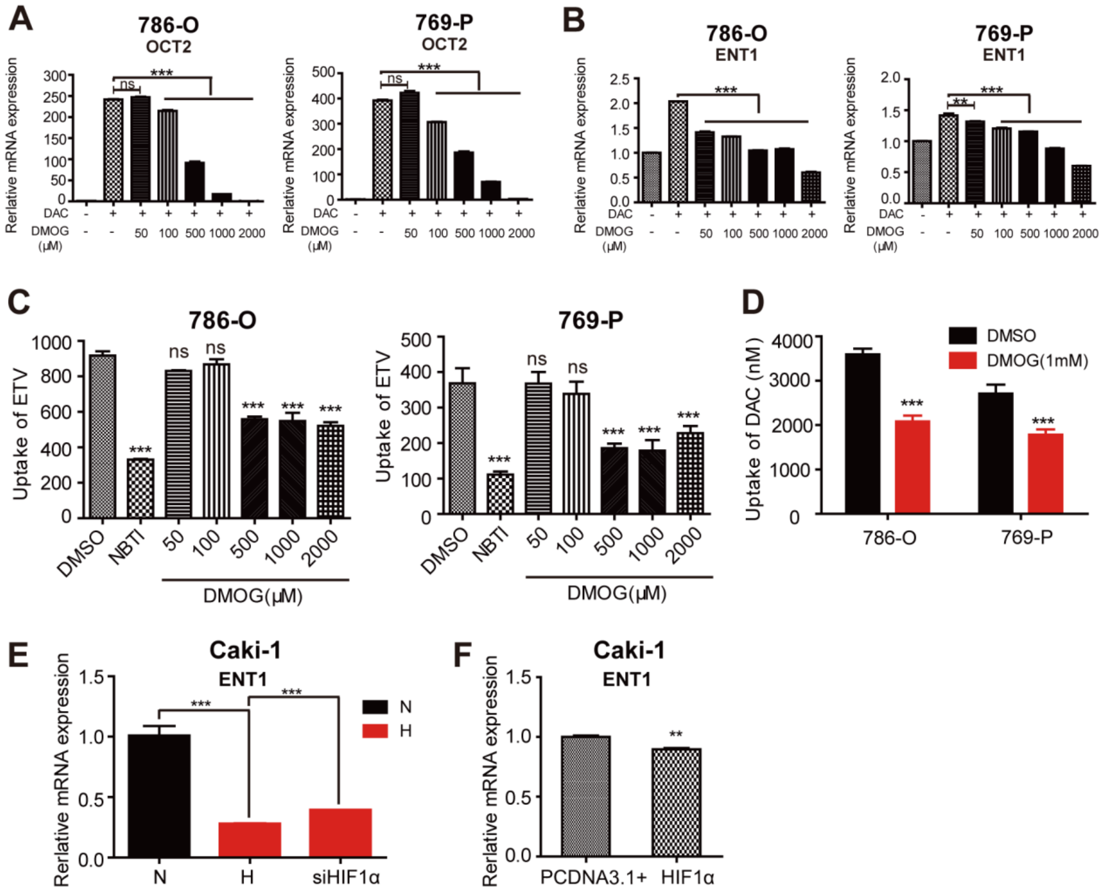

Figure.S6 DMOG treatment-mediated DAC resistance in RCC cell lines under normoxia. (A) Induction ability of DAC to OCT2 was reduced in RCC cell lines (786-O and 769-P) after treatment with DMOG at indicated concentrations. (B) The mRNA expression level of *SLC29A1* was repressed in RCC cell lines after treatment with DMOG at indicated concentrations. Accumulation of entecavir (C) and DAC (D), two substrates of ENT1, was reduced in 786-O and 769-P cell lines after treatment with DMOG at indicated concentrations. The mRNA expression level of SLC29A1 was detected in Caki-1 cells, transfected with specific HIF-1α siRNA under normoxia or hypoxia (E) or transfected with HIF-1α overexpression vector or empty vector in normoxia (F). Oneway-ANOVA analysis was used for

A, B, C, E while student’s *t* test (two-tailed) was used for D, F. The results are expressed as mean ± SEM (n=3). ***P<0.001.

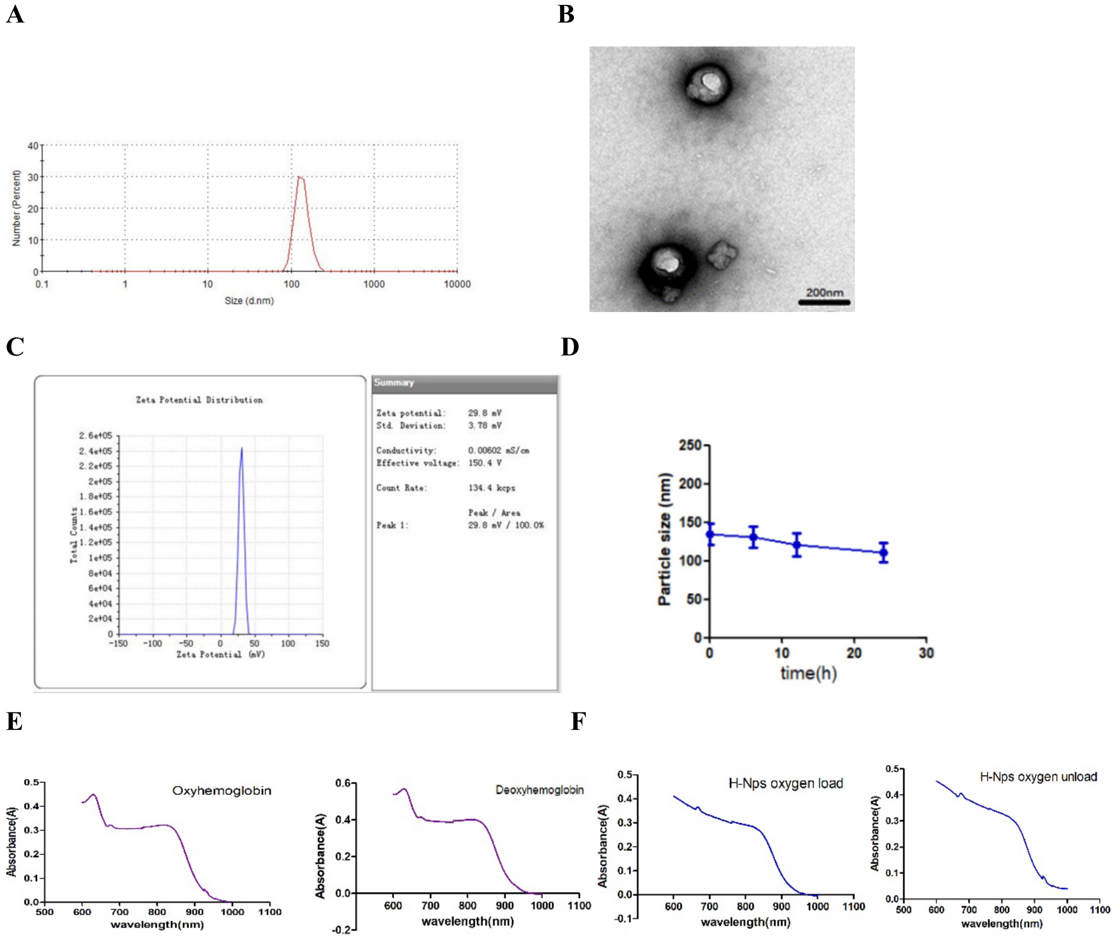

Figure.S7 Characterizations of H-Nps. (A) Particle size distribution of H-Nps 136.5±25.53nm. (B) Transmission electron microscope TEM of H-Nps, negative stained by 2% phosphotungstic acid PTA. (C) Zeta potential of H-Nps in aqueous. (D) Particle size changes of H-Nps in PBS within 24hrs. (E) UV-vis spectrum of oxyhemoglobin and deoxyhemoglobin; oxyhemoglobin has a lower absorbance peak of 0.3228 than deoxyhemoglobin of 0.4165 at 660nm. (F) The UV-vis spectrum of H-Nps saturated with oxygen and unsaturated state; oxygen unload, 0.4016, 660nm; oxygen load, 0.3648, 660nm.

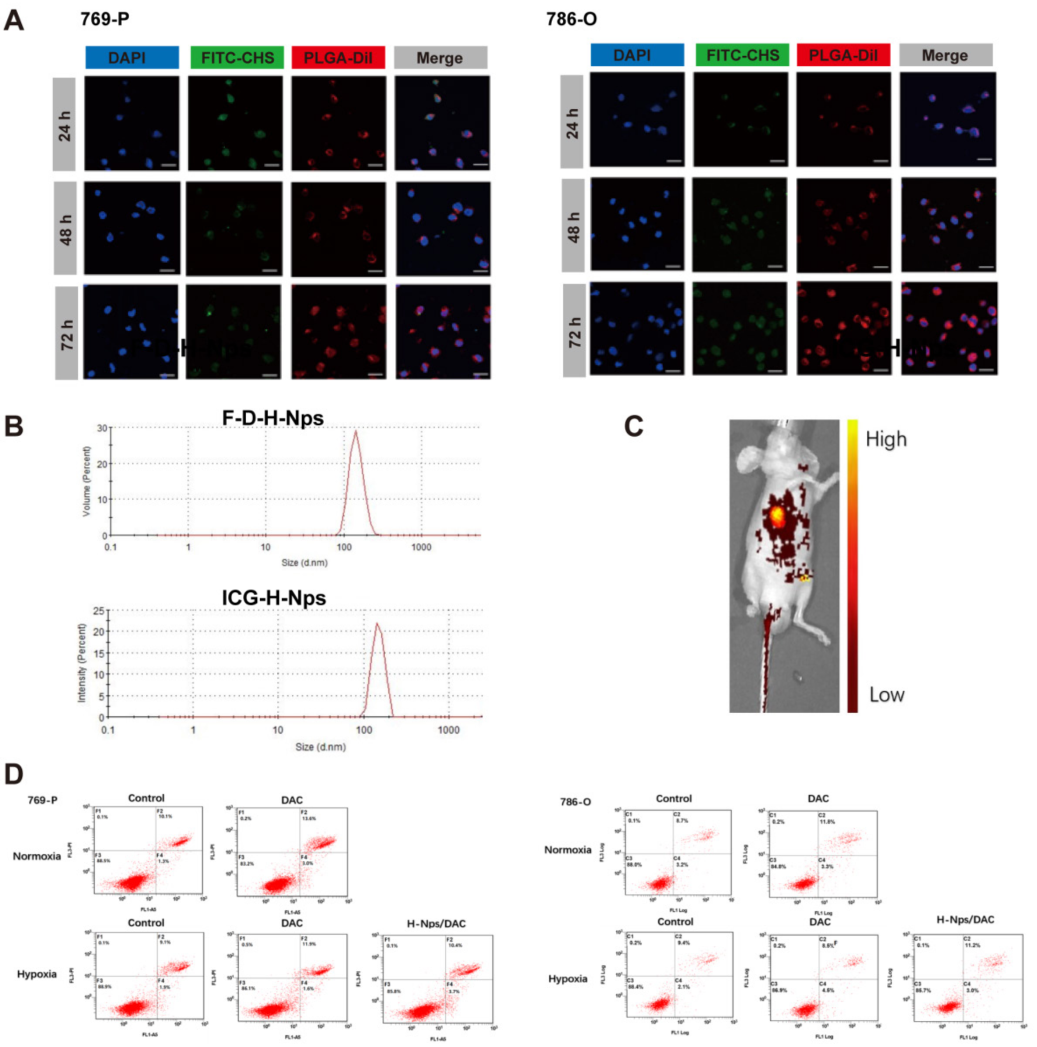

Figure.S8 The cellar uptake and antitumor efficiency study of H-Nps.

(A) 786-O and 769-P were treated with F-D-H-Nps and observed by confocal fluorescence imaging system at 24h, 48h, 72h to investigate intracellular accumulation. Nuclear was stained by DAPI, scalebar=2 μm. (B) The size distribution of ICG-H-nps and F-D-H-nps. (C) A Balb/C nude mouse was injected subcutaneously with 10^7 RCC cells. After tumor formation, the ICG-H-Nps was injected via the tail vein (0.6mg/ml). The fluorescence spectrum of ICG-H-Nps was detected. (D) The apoptotic proportion of RCC cells in different groups was analyzed by flow cytometry using Annexin V-FITC and Propidium iodide dual-staining assay after treatment of oxaliplatin for 48 h.

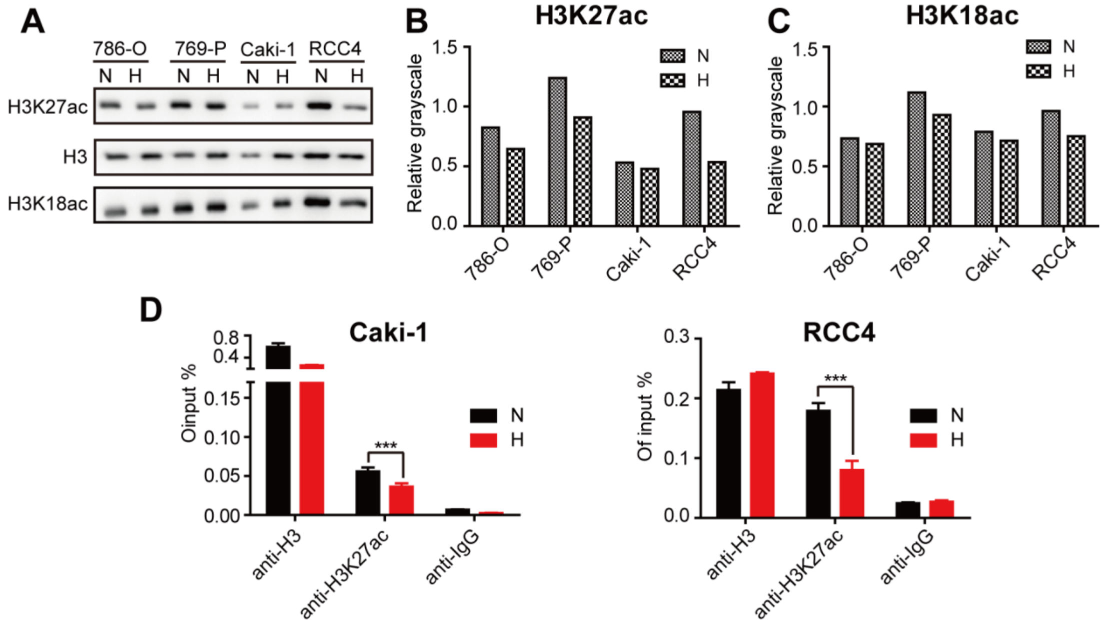

Figure.S9 Hypoxia-mediated modification of histone acetylation for the regulation of OCT2 expression. (A) Four RCC cell lines (786-O, 769-P, Caki-1 and RCC4) were exposed to normoxia or hypoxia for 48 h. Total protein was extracted to determine the global histone modification of H3K27ac and H3K18ac. H3 was used as a loading control of membrane extracts. N, normoxia; H, hypoxia. (B,C) Image J was used for westernblot grayscale analysis. (D) The enrichment of H3K27ac modification level at the OCT2 promoter region in Caki-1 and RCC4 was detected by CHIP-qPCR analysis after exposed to normoxia or hypoxia for 48 h.

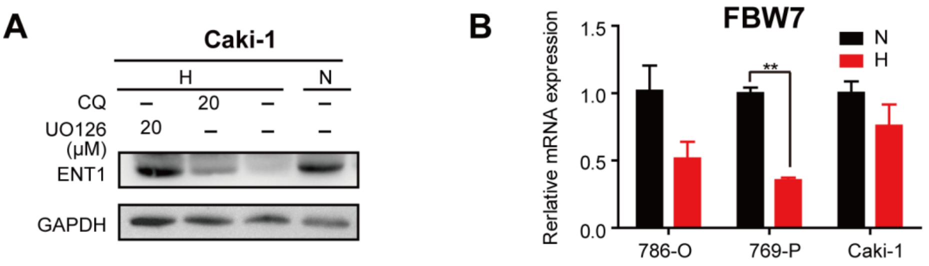

Figure.S10 FBW7-mediated regulation of ENT1 under hypoxia. (A) The protein expression level of ENT1 in Caki-1 cell lines, treated with indicated concentration of lysosome inhibitor chloroquine (CQ) or MEK inhibitor UO126. (B) The mRNA and protein expression level of FBW7 was detected in RCC cell lines, exposed under normoxia or hypoxia for 48 h.

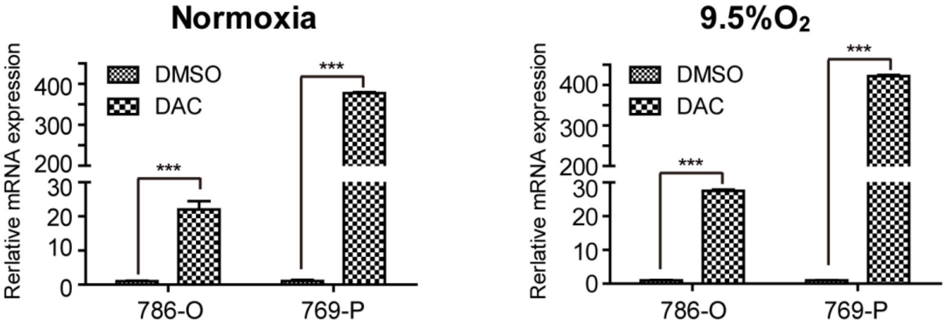

Figure.S11 Induction of OCT2 by DAC under normal renal oxygen concentration. Quantitative RT-PCR analysis of SLC22A2 mRNA transcripts in 2 RCC cell lines (786-O, 769-P) after being treated with 2.5 μmol/L DAC for 72 h in normoxia and normal renal oxygen concentration.

Supplementary tables

Table S1. Primers used in this study

qRT-PCR:

Gene		Primer 5'-3'
OCT2	Forward	AAGAATGGGGATCACAATGG
	Reverse	AGATGTGGACGCCAAGATTC
GAPDH	Forward	AGGTGAAGGTCGGAGTCA
	Reverse	GGTCATTGATGGCAACAA
TDG	Forward	CTTCAGCCCCATAAGATTCCA
	Reverse	ATATTGCACCTCTTGAACGTC
AID	Forward	GCGTGACAGTGCTACATCCTT
	Reverse	TGCGAAGATAACCAAAGTCCAGT
DNMT1	Forward	CAACGAGTCTGGCTTTGAGA
	Reverse	GACACAGGTGACCGTGCTTA
DNMT3a	Forward	CGTTGGCATCCACTGTGA
	Reverse	AATGGTCCTCACTTTGCTGAA
DNMT3b	Forward	AGAGGGACATCTCACGGTTC
	Reverse	GGTTGCCCCAGAAGTATCG
TET1	Forward	TCTGTTGTTGTGCCTCTGGA
	Reverse	CTGGTTTGTTGTCAAAATCTGCCTT
ENT1	Forward	TCTCCAACTCTCAGCCCACCAA
	Reverse	CCTGCGATGCTGGACTTGACCT
ENT2	Forward	ACCATGCCCTCCACCTACAG
	Reverse	GGGCCTGGGATGATTTATTG
CNT1	Forward	ACACTCTCTGAAGTCCACGTTG
	Reverse	AATCCACAGAGGGCAAACGTC
CNT2	Forward	TGGCCTTGTTTGTCATCACCT
	Reverse	TCCTGCAAACACCCATTTCGT

CNT3	Forward	GACATCAACTGCCATCACGTT
	Reverse	TTGGCAACAAGCTATCACCT
DCK	Forward	GGCCGCCACAAGACTAAGGA
	Reverse	CACCATCTGGCAACAGGTTCA
CDA	Forward	CCTGCAGGCAAGTCATGAGAG
	Reverse	ACCATCCGGCTTGGTCATGTA
HDAC7	Forward	CCATGACGACGGCAACTTCTT
	Reverse	TGCTGCGTCATGTATCCAAAAC
HDAC9	Forward	AGTGTGAGACGCAGACGCTTAG
	Reverse	TTTGCTGTCGCATTTGTTCTTT
β-actin	Forward	CATGTACGTTGCTATCCAGGC
	Reverse	CTCCTTAATGTCACGCACGAT

CHIP-qPCR:

Gene		Primer 5'-3'
OCT2	Forward	GGGGCACTACGGGAAGAT
	Reverse	CACCGCAGGGAATCTGAG
ENT1	Forward	GTGTCAGTGCACATCTGCCTGGC
	Reverse	CCTGTCCGCTTCCCCTTTCTAAG

MSRE-qPCR:

Position		Primer 5'-3'
E-BOX	Forward	GACACGGATCCTGGTTCACATCACG
	Reverse	CAGTAATCTTCCCGTAGTGCCCCGA

BSP:

Position		Primer 5'-3'
-358~-26bp	Forward	TGTTGGTTGGAGAATGAGTTTAGTAG
	Reverse	TTCTACACTTCCCAAATACCCTAAA

Table S2. SiRNA and probes used in this study

**Name**	**Sequence (5'-3')**
siTET1-S	AGAUGGCCGUGACACAAAUTT
siTET1-A	AUUUGUGUCACGGCCAUCUGC
siHIF-1α-S	CUGAUGACCAGCAACUUGAdTdT
siHIF-1α-A	UCAAGUUGCUGGUCAUCAGdTdT
siENT1-1-S	CGGCCACUCAGUAUUUCACdTdT
siENT1-1-A	GUGAAAUACUGAGUGGCCGdTdT
siENT1-2-S	ACCAAGUUGGACCUCAUUA-TT
siENT1-2-A	UAAUGAGGUCCAACUUGGU-AG
siENT1-3-S	ACCAAUGAAAGCCACUCUA-TT
siENT1-3-A	UAGAGUGGCUUUCAUUGGU-AG
siARNT1-1-S	GGCGUAUCCUGGAUCUAAATT
siARNT1-1-A	UUUAGAUCCAGGAUACGCCCT
siARNT1-2-S	CAGUUUCUGUGAAUAGGCUTT
siARNT1-2-A	AGCCUAUUCACAGAAACUGGG

Table S3. Tissue specimen information

Number	Age	Gender	TNM stage	Subtype
2	33	Male	T1aN0M0	Clear cell renal cell carcinoma
3	56	Female	T1bN0M0	Chromophobe cell renal cell carcinoma
4	52	Male	T1bN0M0	Clear cell renal cell carcinoma
6	53	Male	T3N0M0	Clear cell renal cell carcinoma

7	52	Male	T1aN0M0	Clear cell renal cell carcinoma
8	50	Male	T1bN0M0	Advanced renal cell carcinoma
9	40	Female	T2N0M0	Advanced renal cell carcinoma
10	64	Male	T1bN0M0	Clear cell renal cell carcinoma
11	70	Female	T1bN0M0	Chromophobe cell renal cell carcinoma
12	48	Female	T1aN0M0	Clear cell renal cell carcinoma
13	28	Male	T1aN0M0	Papillary renal cell carcinoma
14	53	Male	T1bN0M0	Chromophobe cell renal cell carcinoma
16	52	Female	T1aN0M0	Clear cell renal cell carcinoma
17	58	Male	T1aN0M0	Clear cell renal cell carcinoma
18	71	Female	T1aN0M0	Clear cell renal cell carcinoma
20	54	Male	T1aN0M0	Clear cell renal cell carcinoma
25	67	Male	T1aN0M0	Clear cell renal cell carcinoma
27	57	Male	T1aN0M0	Clear cell renal cell carcinoma
28	67	Male	T1aN0M0	Clear cell renal cell carcinoma
29	57	Male	T1aN0M0	Clear cell renal cell carcinoma
30	50	Male	T1bN0M0	Clear cell renal cell carcinoma
32	44	Female	T1aN0M0	Clear cell renal cell carcinoma
33	50	Male	T1aN0M0	Clear cell renal cell carcinoma
34	55	Male	T3N0M0	Clear cell renal cell carcinoma
36	50	Female	T1aN0M0	Clear cell renal cell carcinoma
37	47	Female	T1aN0M0	Clear cell renal cell carcinoma
38	56	Male	T1bN0M0	Clear cell renal cell carcinoma
42	42	Male	T1bN0M0	Clear cell renal cell carcinoma
43	50	Male	T1aN0M0	Clear cell renal cell carcinoma
44	52	Male	T1aN0M0	Clear cell renal cell carcinoma
45	36	Male	T2bN0M0	Clear cell renal cell carcinoma
47	33	Male	T1aN0M0	Clear cell renal cell carcinoma
50	52	Male	T1aN0M0	Clear cell renal cell carcinoma

51	50	Male	T1bN0M0	Clear cell renal cell carcinoma
52	56	Male	T2aN0M0	Clear cell renal cell carcinoma
53	59	Female	T2aN0M0	Clear cell renal cell carcinoma
54	42	Male	T1bN0M0	Clear cell renal cell carcinoma
56	64	Male	T1aN0M0	Clear cell renal cell carcinoma
57	54	Male	T2bN0M0	Clear cell renal cell carcinoma
58	59	Female	T4N0M0	Clear cell renal cell carcinoma
